# Genetic algorithms reveal profound individual differences in emotion recognition

**DOI:** 10.1073/pnas.2201380119

**Published:** 2022-11-02

**Authors:** Nicola Binetti, Nadejda Roubtsova, Christina Carlisi, Darren Cosker, Essi Viding, Isabelle Mareschal

**Affiliations:** ^a^Department of Psychology, Queen Mary University of London, London, E14NS, United Kingdom;; ^b^Department of Computer Science, University of Bath, Bath, BA27AY, United Kingdom;; ^c^Psychology and Language Sciences, University College London, London, WC1H 0AP, United Kingdom

**Keywords:** facial expressions, emotion recognition, genetic algorithms, machine learning, faces

## Abstract

Emotional communication relies on a mutual understanding, between expresser and viewer, of facial configurations that broadcast specific emotions. However, we do not know whether people share a common understanding of how emotional states map onto facial expressions. This is because expressions exist in a high-dimensional space too large to explore in conventional experimental paradigms. Here, we address this by adapting genetic algorithms and combining them with photorealistic three-dimensional avatars to efficiently explore the high-dimensional expression space. A total of 336 people used these tools to generate facial expressions that represent happiness, fear, sadness, and anger. We found substantial variability in the expressions generated via our procedure, suggesting that different people associate different facial expressions to the same emotional state. We then examined whether variability in the facial expressions created could account for differences in performance on standard emotion recognition tasks by asking people to categorize different test expressions. We found that emotion categorization performance was explained by the extent to which test expressions matched the expressions generated by each individual. Our findings reveal the breadth of variability in people’s representations of facial emotions, even among typical adult populations. This has profound implications for the interpretation of responses to emotional stimuli, which may reflect individual differences in the emotional category people attribute to a particular facial expression, rather than differences in the brain mechanisms that produce emotional responses.

## Significance

We developed a genetic algorithm tool allowing users to refine depictions of facial expressions until they reach what they think the expression reflecting a particular emotion should look like. The tool provides an efficient sampling of expression space, ideally suited for capturing individual differences in emotion recognition. We found that individual differences in the expressions subjects generated via our procedure account for differences in emotion recognition performance. Our discoveries advance research on emotion processing by demonstrating that the same stimulus can elicit different responses in people, which may reflect individual differences in the extent to which it is recognized as an instance of a visual category, rather than differences in brain mechanisms specialized to process affective stimuli.

We communicate our emotional state to one another using facial expressions. Effective social communication therefore requires that the person broadcasting an emotion and the person perceiving that emotion have the same understanding of the relationship between emotional state and facial expression. Indeed, many clinical and preclinical populations (1) show atypical performance on emotion recognition tasks, including individuals with schizophrenia (2–4), depression (5, 6), and right-hemisphere brain damage (7–9).

The historical theory of facial emotion recognition suggests that there is a “core” set of emotion categories (10–13), each associated with a distinct facial expression (14), that are important for the communication of emotional state (15, 16). This theory of the universality of emotions is supported by more recent advances using artificial intelligence, which show that people adopt similar facial expressions in similar social contexts (17). However, the range of facial expressions that are associated with a particular emotional state is not known. Indeed, most cognitive, preclinical, and clinical research on emotion recognition rely on asking people to judge the emotional state represented by a largely arbitrary sample of stereotypical images. This work assumes that atypical responses to these facial expressions indicate atypical affective processes. However, this assumption is not met because we do not know the facial expression(s) that an individual associates with a particular emotion. Each individual may have a unique idea in their mind of how an emotion should be represented in a facial expression. Consequently, we do not know whether atypical responses reflect differences in people’s expected depictions of facial expressions or differences in subsequent affective processes. Distinguishing individual differences in people’s expected facial depictions from individual differences in affect is of critical importance to advance theory and to develop targeted interventions for certain clinical conditions.

Previous work on facial emotion recognition has been limited because the space of possible facial expressions is intractably large and is not amenable to parametric investigation (18). Most images of facial expressions used to probe emotion processing are therefore posed, acted-out, and overemphasized depictions of “prototypes” (12), and most experiments require observers to attach to these images an emotional label drawn from a limited and predefined set (18). These experimental choices artificially constrain the investigation of individual differences (19). More recent data-driven approaches apply reverse correlation methods that can reveal information about people’s expectations of what the stimulus should look like for simple stimuli (20–22) and more complex ones such as faces (23). Indeed, Smith et al. (23) found that people’s expectations (sometimes referred to as internal representations) about faces are informative, unique to each individual, and stable over time. Similarly, the reverse correlation of random movements applied to computer avatar faces reveals information about representations of facial emotions (24, 25). However, the very large number of trials required for reverse correlation methods limit measuring the variability of representations at the population level.

Alternative approaches allow for a more efficient exploration of stimulus space (26, 27). We have shown that genetic algorithms (GAs) can provide an efficient sampling of the space of facial expressions (28) and that the resultant evolved expressions are stable depictions for an individual’s expectations of emotional categories (29). Here, we combine GAs with a photorealistic expression-rendering pipeline (30), allowing a less constrained and finer evolution of facial expressions controlled by 149 expression features. We use the tool to measure the facial expression associated with each of several emotional categories, among a large sample of healthy adults. We then demonstrate that individual differences in these facial expressions predict performance in a standard emotion recognition task. Our findings indicate that performance in these tasks largely varies as a function of the similarity between an individual’s preferred depiction of a facial expression and the test images of facial expressions that are used in a task. This implies that responses to emotional stimuli may reflect the extent to which a stimulus is recognized as an instance of a visual category and/or reflect affective processing of the emotional content of the stimulus. Therefore, tasks involving emotional stimuli potentially tap into both perceptual and affective processes, and our tool may be important in accounting for perceptual differences when examining typical and atypical affective processing.

## Results

### Personalized, Photorealistic Facial Emotional Expressions.

**Fig. 1.**
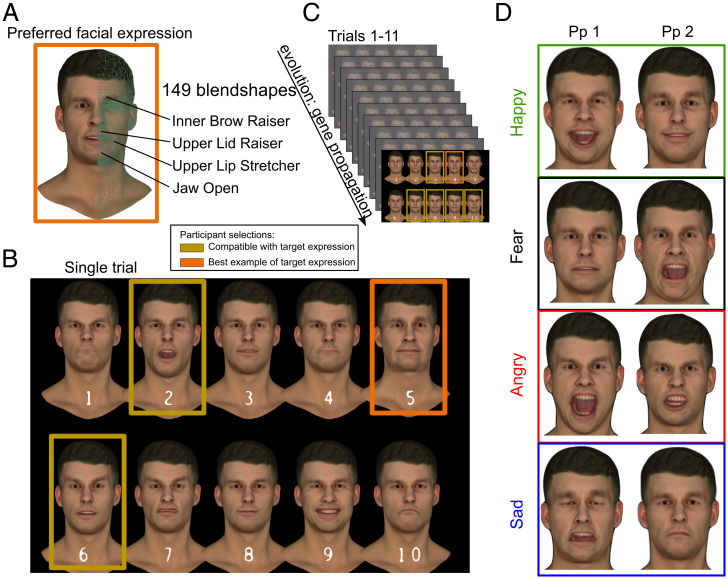
Participants created facial expressions on photorealistic avatars to depict core emotion categories. (*A*) Avatar expressions were controlled through 149 facial expression dimensions. (*B*) A GA evolves expressions through selections and random mutation processes that mimic biological evolutionary mechanisms. Within trials, participants select expressions that match the target expression they are tasked with creating (any number, ranging from 1 to 10 selections) and indicate among these selections the expression that best matches the target expression. (*C*) Across trials, expressions are evolved based on these selections, converging on their preferred facial expression by the 11th trial (10th generation). The final best example selection is taken as the preferred facial expression to the target emotion. This provides insight into the participant’s beliefs of which configuration of facial features reflects an emotional state. (*D*) Examples of preferred expressions evolved by two participants (more examples provided in *SI Appendix*, Figs. S8–S11).

We used photorealistic three-dimensional (3D) avatars to portray facial expressions in a 149-dimensional expression feature space (Fig. 1*A*; *SI Appendix*, *GA Framework*). Each participant created the expression that they believed corresponded to a particular emotion (happy, sad, angry, fear). We used a GA (*Methods*) to allow participants to evolve the faces to reach their preferred expression over multiple trials. On each trial, the participant chose from 10 candidate faces those that resembled the emotion and indicated the expression that provided the best match (Fig. 1*B*). These selections informed the next generation of facial expressions (Fig. 1*C*). After the 11th trial, participants chose the avatar that provided the expression that best matched the emotion (Fig. 1*D*), and we call this final selection their “preferred facial expression” (*SI Appendix* *GA – Pros and Cons* for limitations involved with the GA method).

We found that the facial expressions evolved by participants rapidly converged to a stable position in the multidimensional expression space (*SI Appendix*, Fig. S1 and see ref. 30 for simulation data). Within 7 trials (∼5 min of viewing time), the variance of the faces selected was constant. The GA therefore allowed a rapid and efficient exploration of the multidimensional expression spaces that are necessary to support photorealistic rendering and made it possible to define each of 336 participants’ preferred expressions, for each of four emotions, within the same quantitative space.

In order to gauge how closely the evolved faces matched the target emotion the participant was creating, we asked participants to rate on a Likert scale (1, very poor to 7, very good) how satisfied they were with their preferred facial expression they selected from the final trial. Participants reported a high degree of subjective satisfaction regarding the preferred expressions, suggesting that these expressions provided good approximations of the emotion they were trying to create (*n* = 256; happy: mean of 5.6 ± 1.04, median of 6; fear: 5.42 ± 1.11, 6; angry: 5.76 ± 1.03, 6; sad, 5.38 ± 1, 5.5; *SI Appendix*, Fig. S4). We found a significant difference across emotion categories (χ^2^ (3) = 19.28, *P* < 0.00, nonparametric Friedman test), with significant differences between angry and fear (Z = −3.9, Bonferroni-corrected Wilcoxon signed-rank tests, p corrected = 0.001) and between angry and sad expressions (Z = −3.9, p corrected = 0.001). This suggests that preferred expressions for anger were better matched to people’s idea of anger than those for fear or sadness were matched to their idea of fear or sadness.

### Individual Differences in Preferred Expressions.

Each preferred expression embodies how the participant thinks an emotion should be depicted on the face. We found that each participant converged on unique preferred expressions for the different emotion categories but that there were considerable individual differences within each emotion category. For example, one individual’s preferred expression for fear could be different to another individual’s preferred expression of fear and more similar to their preferred expression for anger (e.g., Fig. 1*D*).

**Fig. 2.**
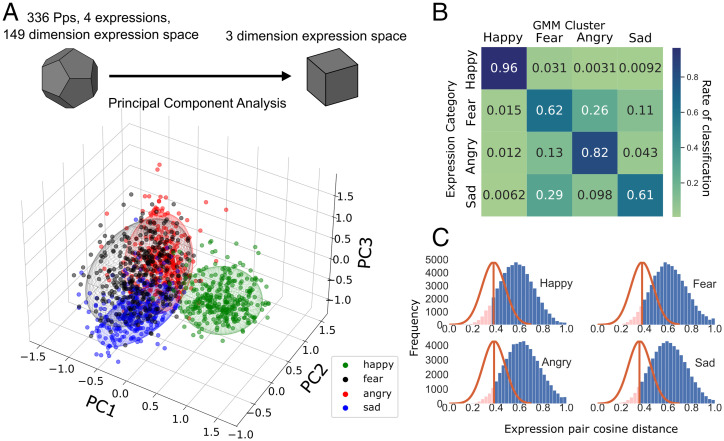
Participants’ preferred facial expression positioning, clustering, and distances in expression space. (*A*) Dispersion of participants’ preferred expressions across the first 3 principal components (PCs) of expression space. Ellipsoids depict the dispersion of expressions per emotion cluster, identified through GMM (radii scaled to encompass 2 SDs per PC). (*B*) GMM confusion matrix depicting the probability of expressions matching the corresponding cluster label and characterizing cluster overlap based on classification rate. (*C*) GA stochastic noise threshold (orange lines) related to the distribution of differences (cosine distance in blendshape space) of all possible participant expression pairings per emotion category (blue histogram). Area of the blue curve above these noise thresholds identify the % of participant expression pairings whose differences exceed variability explained by GA stochastic noise.

We visualized the distribution of preferred expressions by subjecting the 149-dimensional space to principal component analysis. Visual inspection of the space formed by the first 3 principal components (21% of the total variance) suggests that happy formed the most distinct cluster with greater overlap for the other categories (Fig. 2*A*). We subjected the space formed by the first 10 principal components (46% of total variance) to Gaussian mixture modeling (GMM), and identified 4 normally distributed clusters (Fig. 2*A*). We indeed found that happy expressions were the most reliably classifiable (Fig. 2*B*); the GMM was able to identify 97% of happy expressions. We found that anger (82%), and particularly fear (63%) and sad (58%), were less reliably classifiable. Sad was confused with fear in 31% of instances, and fear was confused as angry in 24% of instances. Thus, happy and angry expressions are distinct, but sad and fear are less so.

The individual differences that we find in preferred facial expressions are not explained by stochastic processes in the GA (e.g., noise introduced by random processes in population sample generation). We estimated the difference between each individual’s preferred expression based on cosine distance in expression space (CD; *SI Appendix*, *GA stochastic noise thresholds*), which is a robust distance metric in high-dimensional spaces (30). We calculated the distribution of all possible pair-wise CDs, for each emotion category (Fig. 2*C*, histograms). We compared the observed distributions to ones derived from simulations, which allowed us to estimate the noise that is expected from the GA procedure itself (Fig. 2*C*, red lines; *Methods*). Across 500 iterations, simulated samples were obtained by selecting and evolving expressions aimed at converging on fixed target expressions that corresponded to the average happy/fear/angry or sad expressions. We found that 94% of all observed CDs (happy, 91%; angry, 95%; sad, 95%; fear, 94%) exceed the mean of the simulation (Fig. 2*C*). In other words, the individual differences that we see far exceed those that would arise from noise in the GA procedure.

Given the substantial individual differences in preferred expressions, we sought to examine how recognizable they were in a separate group of participants who had not performed the GA task (*n* = 60). We therefore measured emotion recognition using a forced-choice classification procedure and compared identification performance to performance measured with standard Karolinska Directed Emotional Faces (KDEF). Recognition performance was good with both sets of stimuli, although expressions created with the GA procedure led to lower rates of identification across all categories apart from anger (*SI Appendix*, Figs. S6 and S7). However, this difference in performance may largely reflect the difference in intensity between the two types of stimuli (*SI Appendix*, Figs. S16–S18), supporting the idea that expressions on GA faces are recognizable.

### Features That Distinguish Emotional Expressions.

**Fig. 3.**
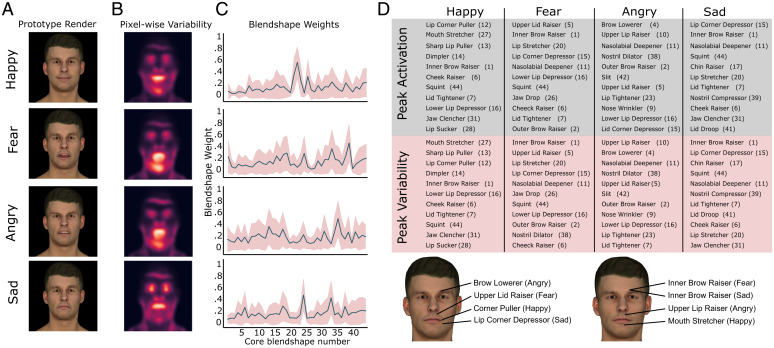
Features that distinguish emotion categories. (*A*) Happy/fear/angry/sad prototypical expression renders based on the average position of preferred expressions in the 149-dimensional expression space (i.e., centroids of emotion clusters). (*B*) Pixel-wise variability (SDs) across all individual expression images providing a visualization of the face areas exhibiting greatest changes across participants. (*C*) Averaged core blendshape vectors (46 blendshapes that can move independently in the GA) reveal category-specific expression feature fingerprints. The shaded region depicts variability in blendshape weights (SD) across participants. (*D*) Expression features (FACS name and AU number) that are most activated (ranked peak blendshape weights) per expression category (gray columns). Expression features that vary the most (ranked blendshape weight variability) per expression category (red columns). The location of most activated and the most variable blendshape within each category are portrayed on neutral avatar expressions.

To estimate a “prototypical” expression for each emotion category, we calculated the average position of preferred expressions in the 149-dimensional expression space (Fig. 3*A*). Averaged blendshape weights (i.e., face feature dimensions) across participants’ expressions reveal category specific “fingerprints.” We identified the dimensions that were most activated within each emotional category and the facial action coding system (FACS) name and associated action unit numbers (AUs) that mostly align with that dimension (Fig. 3*D* and *SI Appendix*, Figs. S12 and S13). For happy expressions, we found strongest activations in lip corner puller, sharp lip puller, cheek raiser, lid tightener, and inner brow raiser movements (AUs 12, 13, 6, 7, and 1, respectively). For fear, these were upper lid raiser, inner and outer brow raiser, lip stretcher, and brow lowerer (AUs 5, 1, 2, 20, and 4, respectively). For angry, they were brow lowerer, upper lip raiser, nasolabial deepener, nose wrinkler, and lower lip depressor (AUs 4, 10, 11, 9, and 16, respectively). For sad, they were lip corner depressor, nasolabial deepener, chin raiser, lip stretcher, and lid tightener (AUs 15, 11, 17, 20, and 7, respectively). Our quantitative estimates show overlap with previous work (12, 31–37) that has generally used a FACS (38) to identify facial components of expressions (*SI Appendix*, Table S1 for comparisons).

Our measurements also allow us to characterize variability in expressions across individuals. Pixelwise variability (SD of pixel intensity) of expression images within each category shows that most variability occurs within mouth, eye (brow/squint), and chin regions (Fig. 3*B*). The variability of blendshape weights across participants (Fig. 3*C*, red shaded region) highlights “blendshapes” along which participants differ the most. We identified the blendshapes with largest individual differences for each emotion category and their corresponding FACS name and AUs (Fig. 3*D* and *SI Appendix*, Figs. S14 and S15). Preferred expressions vary considerably along category specific dimensions, with deviations ranging from 20 to 30% (SD in core blendshape weights) of the maximum extent of motion allowed along these dimensions. For example, participants’ happy expressions might differ in lip corner puller by up to 30% of full activation. We calculated mean weight SD across all blendshapes to provide an overall estimate of variability across participants. Mean SDs showed that participants’ angry expressions vary on average by 21%, fear expressions by 20%, sad expressions by 18%, and happy expressions by 17% of the maximum extent of motion allowed by all blendshapes.

### How Differences in Preferred Facial Expressions Shape Emotion Recognition Performance.

The substantial individual differences in preferred expressions that we report may have repercussions for interpreting the results of standard emotion recognition tasks that rely on prototypical or stereotypical images. If the test facial image is closer to an individual’s preferred expression, they should be better able to categorize it, but if the image lies equidistant between their preferred expressions for different emotion categories, then this is likely to make it difficult for the individual to reliably categorize the expression. By estimating people’s preferred expression and assessing their similarity to the test stimuli, we can account for the perceptual factors that underlie performance in emotion recognition tasks.

**Fig. 4.**
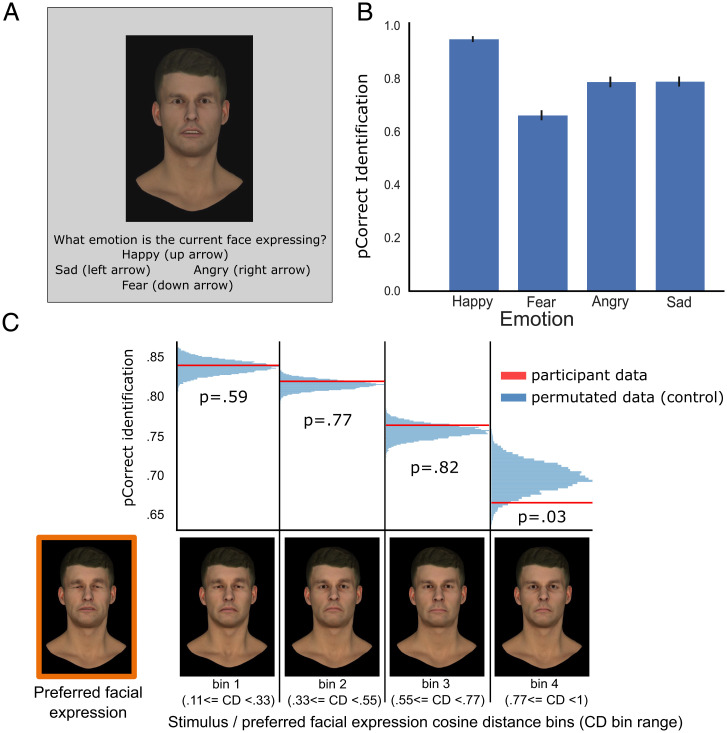
(*A*) Standard choice-from-array expression recognition task requiring participants to label facial expressions evolved by a separate group of participants. (*B*) Probability of correct expression identification as a function of stimulus emotion category, averaged across participants. (*C*) Probability of correct expression identification as a function of test stimulus/preferred expression CD. Within each participant, stimuli were binned based on their CD relative to the participant’s preferred expression for the corresponding emotion category. Higher bin numbers correspond to greater CD (i.e., greater difference) between the participant preferred expression and tested stimulus. To control for central tendency effects, we performed a permutation test comparing participants’ data (red line) against permutated data (blue histograms), in which preferred expressions were iteratively swapped across participants. Performance was significantly (*P* = 0.03) affected only when participants were presented with a stimulus very different from their preferred expression. The expression highlighted in orange (*Left*) shows the sad representation of a sample participant. The 4 stimuli on its right represent sad stimuli samples randomly drawn from bins 1 to 4, arranged from most similar (bin 1) to least similar (bin 4) to the participant’s preferred expression.

To examine this, we tested a subset of participants (*n* = 35) who had previously created expressions with the expressions (happy, fear, angry, or sad) created by a different group of participants (Fig. 4*A*). Averaged emotion recognition performance in this forced-choice task showed comparable performance to the validation data (Fig. 4*B*). We then evaluated emotion recognition performance as a function of the similarity between the test expressions and the perceiver’s preferred expression by binning test expressions based on their difference relative to each perceiver’s preferred expression of the same emotion category (e.g., CD between each sad stimulus and the perceiver’s sad preferred expression; CD between each angry stimulus and the perceiver’s angry preferred expression), and calculated across all emotions of each participant the probability of correct identification per bin.

There is consensus in how people interpret expressions (also evidenced in the overlap of evolved expression features), so the dependence of performance on the distance between test stimulus and preferred expression may reflect deviations from mean category expressions (i.e., a central tendency). To estimate the contribution of the central tendency, we performed a permutation test. Instead of plotting performance as a function of the cosine distance between the test stimulus and the participant’s preferred facial expression, we calculated the distance between the test stimulus and the preferred facial expression of a randomly chosen other participant. For example, participant 1 was assigned the preferred facial expressions created by participant 12. We again binned these cosine distances (collapsed across all emotions, test stimuli, and participants) and calculated the percent correct identification in each of the 4 bins. We repeated this randomization 10,000 times to derive the distribution of simulated percent correct shown by the blue histograms in Fig. 4*C*. The real proportions of correct responses were compared against these distributions to determine whether the performance significantly differed from the central tendency.

We also report, using a different task and set of participants, that expression discrimination performance is also dependent on participants’ preferred facial expressions (*SI Appendix* *Expression discrimination task* for details).

## Discussion

We describe a highly versatile approach that reliably and efficiently captures people’s expectations of how different emotion categories map onto facial expressions. This approach and our results represent a substantive contribution in the following three ways. First, this approach reveals large individual differences in preferred expressions of core emotion categories, with significant overlap between fear and sad categories. These differences in preferred expressions in turn influence emotion recognition, with individual differences in performance explained by the extent to which test stimuli resemble participants’ preferred expressions.

Second, the GA provides the means for disentangling perceptual and emotional processes that underlie recognition performance. Accounting for the similarity between preferred expression and stimulus allows us to isolate perceptual factors that contribute to emotional expression processing. This has profound implications for the interpretation of affective processing in healthy and clinical populations.

Finally, the GA allows for bespoke expression stimuli, tailored to account for variability in expression representations, that can be used in emotion recognition research. With this approach, people can efficiently generate stimuli of facial expressions, bypassing the constraints of tasks that rely on the identification or recognition of a standardized stimuli set.

### Individual Differences in Preferred Expressions of Core Emotion Categories.

The GA allows us to measure the preferred expressions of emotion categories, defined by a unique combination of facial features (vector of blendshape weights). We find some commonalities in core facial features across participants and emotion categories but also substantial individual differences. Averaged expressions reveal the facial movements contributing most to each category, and these movements overlap with AUs reported in previous work (12, 31, 34–37) (*SI Appendix*, Table S1; AUs for happy: 12, 13, 6, 7, 1; fear: 5, 1, 2, 20, 4; angry: 4, 10, 11, 9, 16; sad: 15, 11, 17, 20, 7). However, we also observed considerable variation in evolved expressions (*SI Appendix*, Figs. S8–S11), as evidenced by the spread of emotion clusters in PCA space, with largest weight variability occurring along category-defining blendshapes. Importantly, GA noise accounts for only a small amount of the heterogeneity in expressions. That is, our methodology produces a variable set of preferred expressions (mostly rated as good by the participants after they were created) that, on average, agree with previously documented descriptors of these emotion types. Differences in emotion recognition are well documented between different clinical populations (2–6, 39) and cultural groups (24, 40–43). Here, we show that there exist substantial individual differences in a nonclinical group of adults that may help explain variability in emotion recognition tasks.

How do these individual differences in preferred expressions and the significant overlap between fear and sad categories reconcile with the concept of expression prototypes? According to a prototype framework, instances of an expression vary around a core template (44–46). While people’s preferred expressions of an emotion might vary, these should always be recognizable as instances of a prototype. Alternatively, category representations may vary beyond what would be expected in terms of variations of a core prototype (19, 40, 47), leading to the suggestion of dimensional representations of emotions. Our findings align with this latter view. For example, GMM cluster analysis showed substantial misclassification of fear and sad categories, suggesting that a third of our participant’s preferred expression for sad resemble the average fear expression more than the average sad expression. These findings indicate that an average, prototypical representation of a sad expression does not adequately describe what a substantial percentage of participants define as their preferred expression of that emotion category. The overlap between sad and fear categories is consistent with greater perceptual confusability observed in discrimination tasks (48) and lower recognition performance for fearful expressions relative to other categories (49), as well as these emotions lying next to each other on the emotion circumplex and fear and sad sharing AUs 1 and 4 (38, 50). This might also reflect the higher difficulty of posing fearful expressions that has been previously reported by expressors (51). Misclassifications of sad expressions as angry are also consistent with previous reports of these categories sharing the most characteristic AUs (12).

### Role of Perceptual Processes in Emotion Recognition.

We find that individual differences in preferred facial expressions can explain individual differences in emotion recognition (Fig. 4) and discrimination (*SI Appendix*, Fig. S19), providing a means of accounting for perceptual processes in response to emotional stimuli. For example, research dating back to the 1980s has shown that clinical conditions such as schizophrenia (2–4) and depression (5, 6) are characterized by atypical responses to emotional stimuli. However, more recent studies have challenged these findings by showing weaker (52) or nonsignificant (1, 53, 54) impairments in performance in these clinical populations. These conflicting findings have raised questions regarding the extent to which recognition performance reflects more generic cognitive and/or perceptual impairments (1, 55, 56), but control tasks designed to examine this do not probe visual representations of an expression category.

The most direct way of addressing this question is by measuring people’s preferred expressions for different emotion categories and accounting for their similarity to tested stimuli. The GA approach allows us to estimate these preferred expressions in a matter of minutes per emotion category (4 to 5 min for 10 generations, although convergence was achieved by the 6th/7th generation). In our earlier implementation of the GA system, we found that participants’ preferred expressions were stable after a 4- to 6-wk interval (29). Our expression recognition follow-up experiment was run on average 9 to 11 weeks after the GA expressions were evolved, suggesting an even wider window of response stability. We show that emotion recognition performance improves with increasing similarity between test expression stimuli and the participants’ preferred expression. These results have profound methodological and conceptual implications for emotion recognition research. Measures of test stimulus/preferred expression similarity can be explicitly modeled as a covariate when testing differential performance in tasks involving the presentation of emotional expression stimuli. Accounting for test stimulus/preferred expression similarity allows us to specifically link differences in performance between groups (or between conditions) to affective responses elicited by the emotional content of stimuli, removing perceptual matching confounds.

More generally, emotion processing research should make a significant step forward from methods and stimuli that measure (and constrain) performance relative to fixed stereotypes, favoring approaches that account for the diversity and richness of expression representations in the population (19). Measuring and modeling individual’s preferred expressions capture nuances of expression variability, as well as directly assess perceptual processes at the core of emotion decision making.

It is worth highlighting that although our method provides a means of quantifying the role of perceptual factors, it is agnostic to the influence that conceptual factors may have on the creation of these expressions. There is evidence that people with reduced knowledge of emotional concepts have reduced sensitivity in emotion recognition tasks (57) and that conceptual knowledge influences the perception of facial expressions (58). Our task involves participants associating a word label (e.g., fear) with their conceptual knowledge of the emotion to guide their creation of facial expressions. It is likely that participants vary in their conceptual knowledge of the emotion that may influence the individual differences in preferred facial expressions that we measure. Future studies could use the GA toolkit to help disentangle these factors by combining it with tasks that measure conceptual knowledge and correlating how expression clustering relates to semantic clustering.

### GA-Evolved Stimuli: Applications in Emotion Processing Research.

The GA approach allows people to generate ready-to-use facial expressions. It bypasses posed expression databases that typically portray overemphasized expressions (12). Additionally, the GA can be used as a response method, for example by evolving expressions that depict the emotion a given person would feel in response to some event. Although we found that rates of identification were slightly lower across all categories—apart from anger—when participants were tested with GA preferred expressions compared to Karolinska (KDEF faces, the GA faces were still highly recognizable. There are a number of factors that might underlie this difference with KDEF faces. Firstly, this could partly reflect the artificial nature of GA stimuli or limitations in the evolutionary algorithm in generating expressions on par with real actor portrayals (despite high participant subjective ratings of evolved expressions). Although this is possible, a greater difference between the two stimuli sets is that KDEF expressions are of a higher expression intensity (intensity comparisons in *SI Appendix*, Fig. S16–S18), are highly posed, and are overemphasized. Recognition performance in humans and machine classifiers is typically higher with heavily posed stimuli, as opposed to more subtle (less intense) and variable real-world expressions portrayed in spontaneous databases (59–61). A final point to consider is that the current GA and KDEF stimuli are built with different goals in mind. Our participants were instructed to evolve stimuli that reflect how they think an emotion should be depicted on the face, whereas in posed databases, actors are requested to express (i.e., signal) strong and clear emotions (62). Therefore, higher recognition for KDEF stimuli could potentially reflect a greater emphasis placed on the deliberate communicative intent of these posed expressions. This may not be an accurate reflection of the degree of communicative content in naturally displayed (as opposed to posed) emotional expressions.

While standardized databases provide stimuli that are recognized by most people, they offer no insights as to why participants might respond differently to these stimuli. Our data show substantial variability in preferred expressions across people, with a measurable impact on emotion recognition. Thus the GA can be used to build bespoke stimuli sets, where participants are presented with stimuli that they had previously evolved or that closely match their preferred depiction of an expression, therefore removing perceptual confounders. Alternatively, performance with bespoke sets can be compared to stimuli that deviate from participants’ preferred depiction of an expression, exploring on an individual basis the tolerance to deviations from a category. Additionally, GA stimuli come with the added advantage of offering rigorous control over positioning/orientation, lighting, context, and identity of 3D models.

The GA approach also provides a means for versatile response methods in emotion recognition studies, without artificially constraining responses to predefined categories. For example, expressions could be evolved to depict the emotion a given person would feel in response to some event or evolve expressions that match some visual target. Importantly, we demonstrate with support vector machine classifiers that emotions targeted by participants can be predicted based on evolved blendshape weights (*SI Appendix*, *Predicting emotion category of new evolved expressions*). This implies that while the task might not explicitly constrain responses to predefined categories, these emotions can be inferred through performance-based criteria.

## Methods

### Participants and Tasks.

#### GA-evolved expressions.

We recruited 336 participants to evolve expressions with the GA toolkit. A group of 293 participants (age = 28 ± 9.6 y old; age range = 18∼68; gender = 172 male, 117 female, 1 nonbinary, 3 prefer not to say) were recruited through the Prolific online recruitment platform (https://www.prolific.co/). Because of the Covid pandemic that precluded in-person testing, these participants liaised with an experimenter through Prolific’s anonymized messaging system and ran the GA experiment remotely from the experimenter’s computer through Google’s remote desktop client (https://remotedesktop.google.com/). In order to control for stimulus presentation conditions, we additionally collected 43 participants (age = 21.5 ± 4.9 y old; age range = 18∼36; gender = 7 male, 36 female) recruited through the SONA platform to evolve expressions under controlled laboratory conditions at Queen Mary University. Given that no substantial difference was observed in expressions evolved by these two groups (online/laboratory; *SI Appendix*), we merged these datasets.

Participants evolved expressions of a 3D male avatar face, selecting stimuli that depicted expressions of happiness, fear, anger, and sadness, across separate counterbalanced blocks. On the first trial (initialization), participants were presented with 10 random expressions (i.e., procedurally generated, *SI Appendix*, *GA – effect of initialization expressions*). On each trial within a block for a given target emotion, participants selected which among 10 expressions resembled the target expression they were trying to create, with no constraint on the number of selections allowed. Participants also additionally flagged a single expression among their selections that provided the closest match to the target expression. On the final iteration, they selected only one face that was their preferred facial expression. Expressions were presented on two rows, with a number label (1 to 10) beneath each expression for reference. For participants tested in the laboratory, at the viewing distance of 57 cm, an individual face subtended ∼2.5 by 3.5 degrees of visual angle. Selections were made in a separate response window by clicking on radio buttons corresponding to each expression number in the display and by typing the number of the expression that best captured the target emotion in a text field. Expressions were evolved across trials (iterations) by a GA, converging on the target expression by the 10th iteration. Avatar expressions were controlled through 149 face motion dimensions (blendshapes), which approximate facial muscle actions as captured by FACS (38). The number of independent dimensions (that can move independently in the GA) is smaller, resulting in 46 core blendshape units. We provide an in-depth description of the avatar blendshape rig, the GA operators enabling user-guided expression evolution, and metrics for a comparison of generated samples in an archived technical report (30). At the end of each emotion category block, we asked participants to rate on a Likert scale (1, very poor to 7, very good) how closely the preferred expression managed to faithfully capture the target emotion. The experiment lasted on average 60 min. Participants were compensated at a rate of £7.50/h. This study was approved by Queen Mary University Research Ethics Committee (QMERC2019/81) as well as University College London Research Ethics Committee (BUCNI-BBK-16-002) and was in agreement with the local research guidelines and regulations. Compensation rates and ethics apply to all experiments below. An analysis of GA expressions was run via custom code written in Python 3.8.5.

#### GA stimuli external validation.

We recruited two separate groups of 60 participants each (group 1: mean age = 27.4 ± 10.1 y old; age range = 18∼66; gender = 34 male, 26 female; group 2: mean age = 29.8 ± 9.9 y old; age range = 18∼60; gender = 37 male, 23 female) that had not been previously tested with the GA system to validate GA expression stimuli through the Prolific online recruitment platform. On each trial, participants in group 1 viewed a happy, fear, angry, or sad expression stimulus, belonging to either the GA stimuli set (randomly selected from the 293 Prolific expression set) or to the KDEF database (only front-facing happy, fear, sad, and angry expressions, randomly selecting between male and female identities) (62). Emotion category and stimulus set were randomly selected across trials, with 4 emotions × 2 stimulus sets × 35 samples, totaling 280 trials per participant. Group 2 performed the same task, with the only exception that GA and KDEF stimuli were blocked separately. This was done in order to control whether grouping of identities (single identity in GA vs. multiple identities in KDEF) might affect results. In group 2, expressions were presented at the center of the screen, with faces measuring on the testing computer ∼4.5 by 9.5 degrees of visual angle. Beneath the stimulus, a text prompt asked participants to indicate the emotion category the stimulus belonged to with a keyboard arrow press. Responses were not subject to time limits, and participants were instructed to favor accuracy over speed. The experiment lasted on average 20 min.

#### GA expression categorization.

A random subsample of participants (*n* = 35; mean age = 28.4 ± 11.1 y old; age range = 18∼68; gender = 21 male, 13 female, 1 prefer not to say) that had previously evolved expressions through the GA was asked to categorize (happy, fear, angry, or sad) expressions constructed by a separate group of participants. Participants were recruited through Prolific, and after reading the information sheet and providing informed consent, they were redirected to an emotion recognition task hosted on Pavlovia. On each trial, participants viewed a happy, fear, angry, or sad GA expression stimulus (constructed by a separate group of 40 participants). Emotion category randomly varied across trials, with 4 emotions × 40 samples, totalling 160 trials per participant. Presentation and response conditions mirrored the external validation study. The experiment lasted on average 12 min.

## Supplementary Material

Supplementary File

## Data Availability

Expression stimuli evolved by participants, the GA tool, custom Python analysis code, and accompanying datasets are available online (DOI 10.17605/OSF.IO/DYFAU) (63).
