# Supplementary material for: Genetic algorithms reveal profound individual differences in emotion recognition
Source: Proc Natl Acad Sci U S A. 2022 Nov 2;119(45):e2201380119. doi: 10.1073/pnas.2201380119 (PMC9659399; doi:10.1073/pnas.2201380119)
Supplement: Supplementary File [file pnas.2201380119.sapp.pdf]

PNAS
www.pnas.org

Supplementary Information for
Genetic algorithms reveal profound individual differences in emotion recognition

Nicola Binetti, Nadejda Roubtsova, Christina Carlisi, Darren Cosker, Essi Viding and Isabelle Mareschal

Nicola Binetti
Email: nicolabinetti@gmail.com

This PDF file includes:

Supplementary Methods
Figures S1 to S19
Tables S1
SI References

GA Framework

*Evolution of preferred expressions with the GA***.** The GA allows users to evolve photorealistic 3D meshes of facial expressions, through a combination of gradual refinement and random processes across generations. Random processes can potentially result in anatomically implausible facial configurations, which is however automatically mitigated by the use of corrective mechanisms (see fig 2 in (1)).

Facial stimuli are uniquely defined using vectors of blendshape weights representing chromosomes of genes in the context of a genetic algorithm. In the process of the genetic evolution of facial stimuli, through repeated visual sample assessment, the participant refines the latent quantitative representation. Specifically, evolution by a genetic algorithm constitutes repeated selection of favourable samples from iteratively refined populations, whereby the refinement is driven by the selection of prior choices. At the same time inherent randomness in the GA through, for example, its mutation and population boosting operators enable facial dynamics space exploration preventing premature convergence.

Each time the tool is initialized, a protocol-generated set of 10 expressions are displayed, involving random generation of two expressions per emotion type (happy, sad, angry and fearful), one arbitrary expression and the neutral expression to compose the initial ten faces. This initialization approach casts a net wide enough to allow proper exploration of the space and avoid premature convergence. Specifically, we initially generate faces from gene pools (sets of blendshapes) characteristic of each type of emotion as well as random faces, seeding enough diversity into the population to enable free space exploration for different emotion types.

On each iteration of the GA, the user selects from the population a number of expressions most similar to some internalized target. Among an unconstrained number of selections, one (elite) face is selected by the participant as the best and there is no further relative fitness ranking of the remaining selected samples. The elite is guaranteed to propagate unchanged to the updated population to exert sufficient selection pressure in the GA. The manner and extent of gene propagation of the non-elite selections can vary and are stochastically governed. Specifically, the two mechanisms for gene propagation are averaging, and the tandem of cross-breeding and mutation. The formal definitions of these operators in the genetic algorithm are given in(1). In simple terms, through averaging the mean of two or more blendshape vectors is propagated to the next population. Cross-breeding and mutation on the other hand involves substitution of randomly selected weights of one chromosome by those of another (“cross-breeding") and the subsequent assignment of new random values to a fixed number of arbitrary genes in the chromosome (“mutation"). Finally, to maintain diversity and avoid premature convergence, the population at each iteration is boosted by 40 % (4 out of 10 samples) insertion of novel samples completely uncorrelated to prior user selections. After calculating when the process plateaus, we chose to terminate the iterative process after 10 iterations with the final (preferred) face being the evolved expression approximating the emotion being created. These measures (stimulus positioning, unrestrained number of selections and population diversity boosting) are designed to ensure an unbiased exploration of expression space, avoid premature convergence, and mitigate risks of serial dependency, where participants’ selections might be based on prior decisions.

*GA stochastic noise thresholds***.** Both protocol-based initialisation of the genetic algorithm and its key population refining processes of mutation, cross-breeding and averaging involve sampling the uniform random distribution. Due to this stochastic element in the evolutionary process, the final evolved face will vary, even given the absolute consistency of the person’s targeted expression. We call this variance that is inherent to the generation process itself genetic algorithm noise. Since the stimuli have a quantitative representation as blendshape vectors, we can simulate genetic evolution to quantify the noise, which provides a threshold in user data analysis. Any difference in excess of the threshold in user-generated distributions can be deemed significant i.e. unlikely to have arisen from the stochastic nature of the generation mechanism itself. The simulation relies on replacing user assessment by a metric comparing population samples to a target that represents

average stimuli of happy, sad, fearful and angry, derived from participant testing. Cosine distance was used to quantify difference of expressions given that it provides a reliable metric for high-dimensional sparse vectors such as the blendshape representation. The cosine distance (CD) is defined as:

$$CD(\alpha_1, \alpha_2) = 1.0 - \frac{\alpha_1 \cdot \alpha_2}{\|\alpha_1\|\|\alpha_2\|}$$

where α_1 and α_2 are the two blendshape vectors being compared

Through 500 simulated iterations, the mean and variance of the inter-sample cosine distance over all combinations of independent final elites in the simulated distribution quantify genetic algorithm noise as the only source of variation.

GA convergence - simulations. We performed simulations to characterize the convergence of the GA. The simulations were aimed at evolving expressions that best matched targets of variable complexity (1,3,12 or 125 active blendshapes), using cosine distance as the relative fitness function. Across 11 iterations each simulation selected expressions “compatible” with the target expression (flagging the “best” example amongst the selection), with the number of selections mirroring average numbers operated by participants within iterations (see Fig. S3, A). Within each iteration, we obtained a distribution of cosine distance errors between the “best” example and the target expression (Fig S1). Simulations showed that shifts in the mean of these error distributions become progressively smaller across iterations, converging by the 11th iteration (i.e. approximately at iteration 7~8, which mimics participant convergence data shown in Fig S2). We also show the target next to the expression flagged as the “best” example on the final iteration, providing visual evidence of the framework’s convergence.

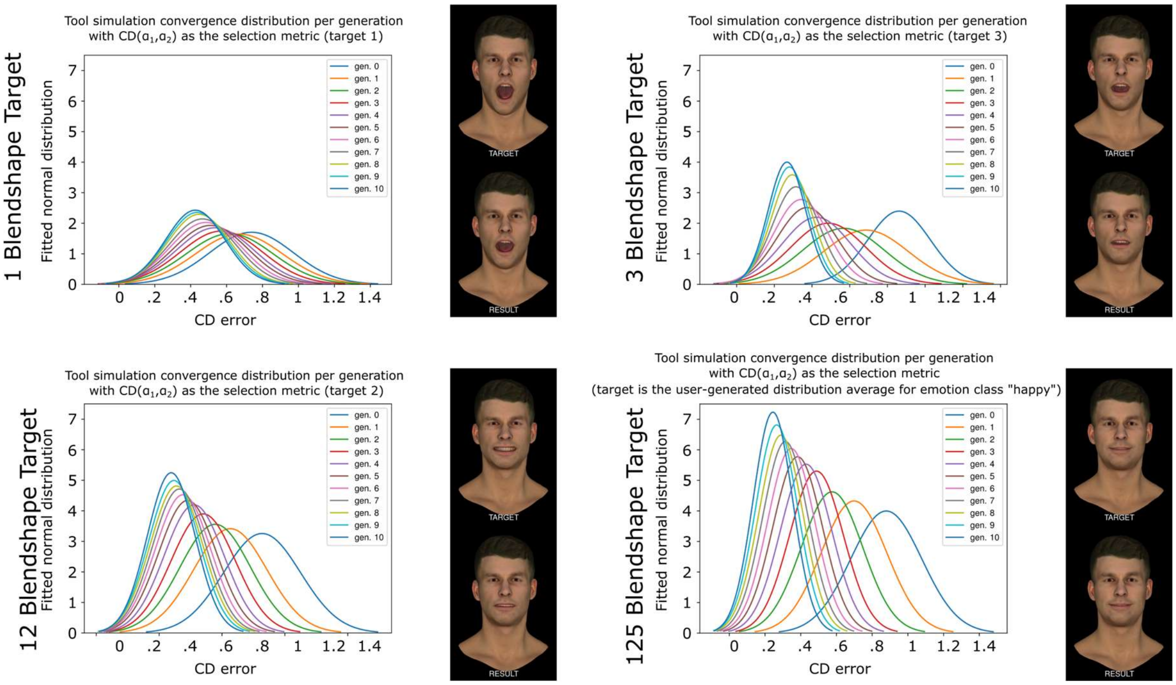

Fig S1: Simulated EmoGen convergence using Cosine Distance (α1; α2) as the selection metric for targets of increasing complexity defined by the number of non-zero weight blendshapes (1,2,12, 125 blendshapes). Also shown for each target are the average faces converged to in the final generation. From Fig 18 in Roubtsova et al. (2021).

GA convergence – evidence from participant data: participant data also empirically suggests GA convergence on the participants' selections. Firstly, in a previous study, participants were asked to evolve the same expression on three separate occasions (2). We observed that participants were systematic in evolving expressions that depicted their preferred facial expressions of emotions. This was evidenced by lower within-subject variability than between-subject variability in the expressions created. Secondly, in our current study, participants rated how closely evolved expressions captured the depiction they had in mind. These ratings showed a high level of satisfaction, suggesting that evolved expressions provided good approximations of the participants intended facial depiction. Finally, and most importantly, these evolved expressions explain participants' identification of emotion categories (as shown in Fig 4 C), which provides evidence that these expressions capture processes that drive expression recognition behavior.

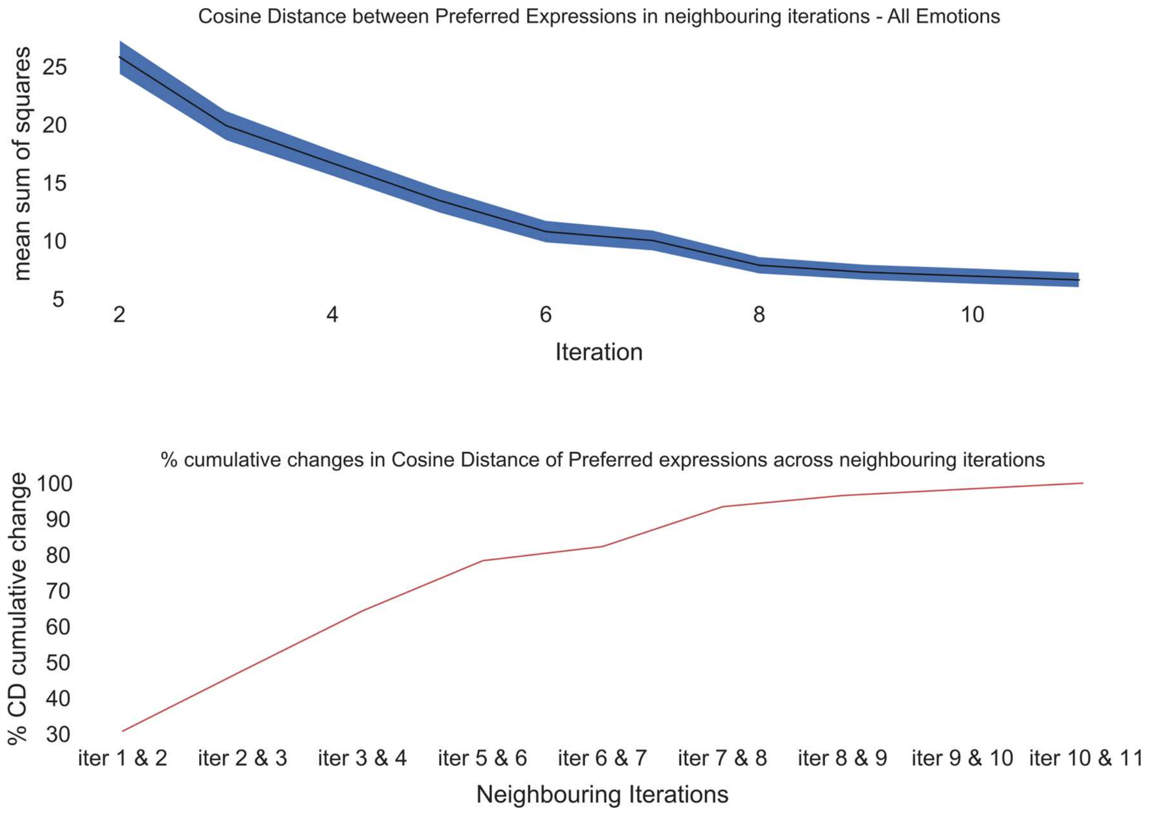

Fig. S2. Differences in expressions across iterations of the GA (Top panel). Within each GA iteration participants must indicate one expression ("preferred expression") amongst all the expressions they selected that best captures the target expression. Across successive iterations we can calculate how much the preferred expressions have changed based on their distance in expression space. We used Cosine Distance (CD) which provides a reliable metric for comparison of sparse high-dimensional vectors such as the blendshape weight representation (1). The plot depicts the distance of preferred expressions on a given iteration relative to the previous iteration (sum of squares of CDs between expressions, averaged across subjects), showing progressively smaller differences in expressions throughout iterations, plateauing approximately around iteration 8 (generation 7). For a more in-depth analysis on convergence with the GA system, see (1). **Cumulative sum of differences in ideal expressions across iterations of the GA (Bottom panel).** Cumulative sum of difference (CD) in expressions across neighbouring iterations, expressed as a % of the sum total of differences across all iterations. Consistent with the above plot, this suggests a plateauing of differences near the 8th iteration**.**

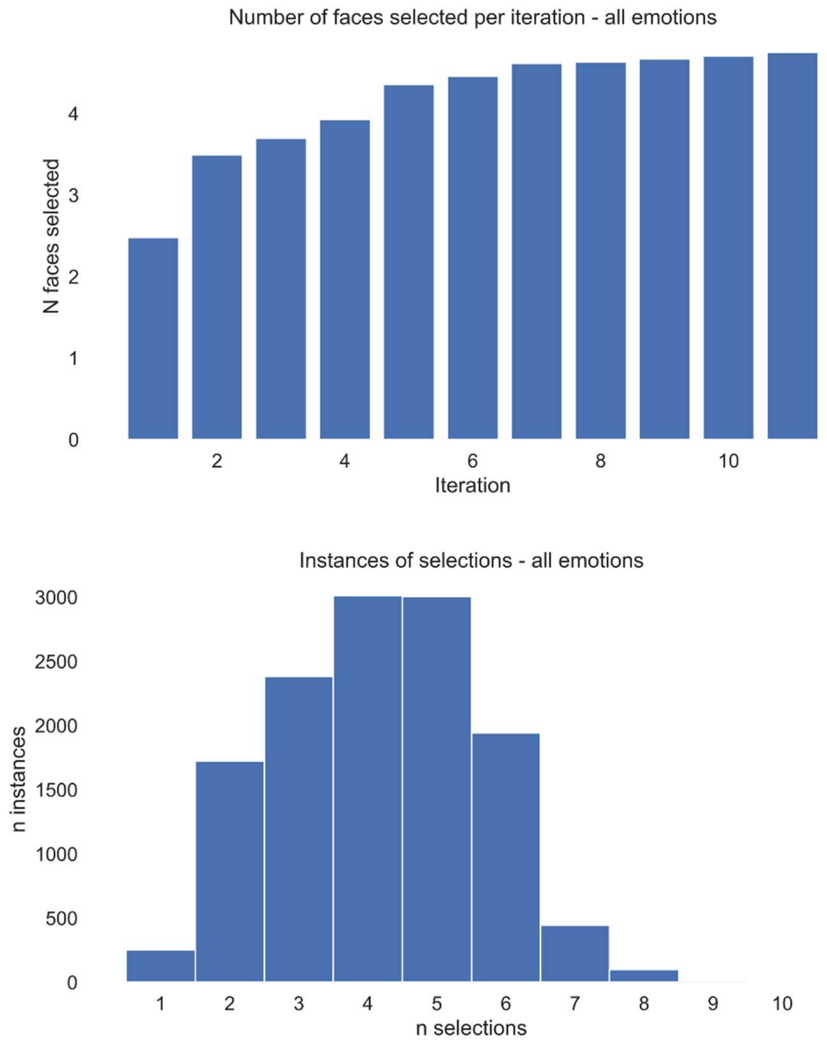

Fig. S3. Participant selections of GA evolved expressions. Top panel: Average number of expressions selected per GA iterations. On each iteration of the GA, participants can select as many expressions (between 1 and 10) that have some resemblance to the target expression. The barplot depicts average number of selections within each GA iteration, showing that the number of expressions selected plateaus to 5 selections by the 7th iteration. This shows that upon initialization, a smaller number of expressions tend to be recognized as similar to the target expression; as expressions are evolved across iterations, the number of expressions indicated as being similar to the target progressively increase, plateauing to 5 selections on average. Bottom panel: Frequency of number of selected expressions across participants.

GA – effect of initialization expressions:

By relying on procedurally generated sets of starting expressions on the 1st trial, we potentially introduce an additional source of noise since the selection of the initial seed is known to impact these search algorithms. However, random starting positions are beneficial as they provide greater flexibility for the GA to explore different areas of expression space. Given that we wanted to capture nuanced differences between participants' depictions of expressions, we opted for the latter so as to not constrain the algorithm in the exploration of expression space. We also wanted to avoid systematically biasing participants in expression space which is a possibility when using a fixed starting configuration. By using procedurally generated starting configurations we essentially treat starting configuration as an additional source of noise in the GA. Importantly the GA noise threshold shown in figure 2, which was compared against individual differences in evolved expressions, is produced by simulated data using random starting configurations, thus accounting for noise introduced by procedurally generated initialization.

The effect of starting configuration (fixed Vs procedurally generated) was assessed through simulated data aimed at evolving expressions across 10 generations that best matched a fixed target expression, using cosine distance as the relative fitness function (as described in SI GA convergence - simulations). We compared the effect of Fixed (Fixed set of 10 faces in the 1st trial across all simulations) or Procedurally generated starting configurations (variable set of 10 expressions on the 1st trial across simulations) through 500 simulations. Comparing final generation distributions, the convergence statistics (cosine distance error mean and standard deviation $\mu \pm \sigma$) were similar for both initializations: 0.47 ± 0.162 and 0.43 ± 0.164 for fixed and protocol-generated initializations respectively (full details can be found in (1)). While this difference was significant, also considering the large number of samples ($t(998)=3.8$, $p<.001$), the effect size was small (Cohen's $d=.24$). Therefore, starting configuration has a negligible impact on final evolved expressions. However, and importantly the GA noise threshold shown in figure 2 demonstrates that the individual differences between participants are not the result of noise introduced by the GA procedure. These noise thresholds were obtained with simulations using non-fixed starting expression configurations that account for noise resulting from procedurally generated initialization

GA – Pros and Cons:
The GA is an efficient search mechanism, we outline below some of its pros and cons
"Pros:
-We contend that some bias is advantageous as we 'want' people to move in a certain direction and not completely randomly on each trial to increase efficiency. However, we also avoid forcing people in a particular direction since we introduce 4 novel samples on each iteration uncorrelated with previous selections.
-GAs look to mimic natural selection – so they are biased in a way that mimics that process.
-The starting point (first iteration) can introduce bias, but this is also randomly determined. We also show that even if we get local solutions people end up roughly in the same spot – which is a good thing (people cluster).
-The introduction of randomness is also produced via the mutations, which give people the chance to branch away from initial choices and reduce bias

Cons:
-Trials are not independent, so some bias can be introduced
-The GA may evolve parameters in non-physically realistic ways and we mitigate this with corrective shapes as part of the 149 blendshapes."

A)

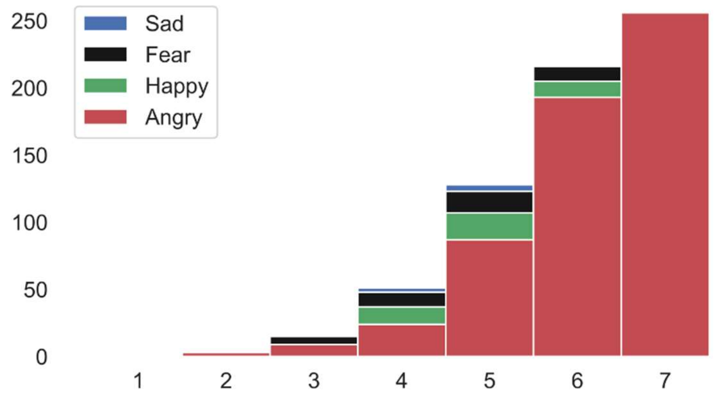

B)

Happy Vs Fear - Z=-2.01, p=0.045, pCorr=0.269
Happy Vs Angry - Z=-2.19, p=0.029, pCorr=0.171
Happy Vs Sad - Z=-1.95, p=0.051, pCorr=0.305
Fear Vs Angry - Z=-3.9, p=0.000, pCorr=0.001
Fear Vs Sad - Z=-.18, p=0.860, pCorr=5.162
Angry Vs Sad - Z=-3.9, p=0.000, pCorr=0.001

Fig. S4. Subjective ratings of preferred facial expressions. A) Distribution of subjective ratings (cumulative sum of Likert scale scores) of how closely the preferred facial expression captured the target expressions (1-very poor / 7-very good). Given that Likert ratings are ordinal, not continuous and have upper / lower bounds, we ran a non-parametric Friedman test, testing the null hypothesis that expression scores come from the same distribution, which revealed a significant difference between emotions ($\chi2(3)=19.28, p<0.00$). **B)** Bonferroni-corrected Wilcoxon signed-rank tests, exploring which emotion pairings contribute to the above result. These show that Angry differed from Sad and Fear expressions, explained by the greater negative skew in the distribution of Angry expression scores.

Predicting emotion category of new evolved expressions.

We used machine learning (Support Vector Machines) to test whether GA evolved blendshape weight vectors could be used to reliably predict the emotion category created by participants, and whether predictions generalized to GA stimuli evolved by different groups of participants. A Support Vector Machine (SVM) classifier was trained to discriminate emotion category based on blendshape weights of faces in a randomly sampled subset of 219 participants. The SVM model was trained by providing each participant's 5 final preferred expressions (i.e. the participant's preferred expressions selected in GA iterations 7 through 11). SVM parameters were optimized in the training set through 5-fold cross-validation, converging on a non-linear Radial basis function (RBF) kernel, C=30 (penalty parameter of the error term), and Gamma=.01 (inverse of the standard deviation of the RBF). The SVM model was subsequently tested by labelling expressions using weight vectors evolved by a separate group of participants (N=74), and correctly identified the emotion type in 86% of cases (binomial test p = 1.4e-37). The classification report below summarizes performance of the classifier and normalized rates of classification.

Accuracy (relative to chance level = 0.25): 0.8633288227334236
Binomial test p = 1.3893856470802085e-37
Classification Report:

	precision	recall	f1-score	support
Angry	0.87	0.84	0.85	370
Fear	0.77	0.78	0.77	370
Happy	0.98	0.97	0.98	370
Sad	0.84	0.86	0.85	368
accuracy			0.86	1478
macro avg	0.86	0.86	0.86	1478
weighted avg	0.86	0.86	0.86	1478

Comparison of GA expressions evolved through online platforms (Online) and expressions evolved in a controlled laboratory environment (Lab). In order to control for stimulus presentation conditions, we collected additional GA data in a controlled laboratory setting. Participants (N=43) evolved the happy, sad, fear and angry expressions using the same laptop in the Lab. We compared expressions between the Online and Lab groups by means of two machine learning approaches.

We first tested whether an SVM classifier trained with data collected online could recognize expressions evolved by participants in the lab. The classifier showed overall comparable performance in the classification of emotions evolved by these two groups: classification accuracy: Online group = 86% (as shown in "Predicting emotion category of new evolved expressions") Vs Lab group = 87% correct classification (see classification report below).

Accuracy (relative to chance level = 0.25): 0.8662790697674418
Binomial test p = 1.3893856470802085e-37
Classification Report:

	precision	recall	f1-score	support
Angry	0.83	0.93	0.88	43
Fear	0.88	0.70	0.78	43
Happy	1.00	0.93	0.96	43
Sad	0.78	0.91	0.84	43
accuracy			0.87	172
macro avg	0.87	0.87	0.87	172
weighted avg	0.87	0.87	0.87	172

The second approach consisted of testing whether an SVM classifier could determine whether evolved expressions came from the Lab or Online group. We supplied the classifier equal numbers of expressions belonging to the two groups (randomly sampling 43 in the Online group). The classifier exhibited chance level performance (53% classification accuracy, binomial test p = .62, see classification report below), suggesting that expressions do not significantly differ between the two groups.

Accuracy (relative to chance level = 0.5): 0.5348837209302325
Binomial test p = 0.6172994135892526
Classification Report:

	precision	recall	f1-score	support
lab	0.47	0.62	0.53	37
prol	0.62	0.47	0.53	49
accuracy			0.53	86
macro avg	0.55	0.55	0.53	86
weighted avg	0.56	0.53	0.53	86

We also collected subjective Likert ratings of how satisfied a subset of these participants (N=31) were with the expression the GA converged on. Below we present the Likert ratings and Kruskal-Wallis test results comparing scores between the Lab and Online groups, for each emotion type.

A) Subjective ratings of GA expressions evolved by 32 participants in the laboratory (Lab group).

Mean scores per category:
Happy 5.597656
Fear 5.417969
Angry 5.765625
Sad 5.382812

std scores per category:
Happy 1.045000
Fear 1.111512
Angry 1.034441
Sad 1.146469

B) Kruskal-Wallis test results (Bonferroni corrected) comparing subjective ratings between Lab and Online groups, for each emotion type.

Lab Vs Online Happy - Statistics=0.000, p=0.999, pCorr=5.994
Lab Vs Online Fear - Statistics=0.168, p=0.682, pCorr=4.090
Lab Vs Online Angry - Statistics=0.231, p=0.631, pCorr=3.786
Lab Vs Online Sad - Statistics=1.362, p=0.243, pCorr=1.459

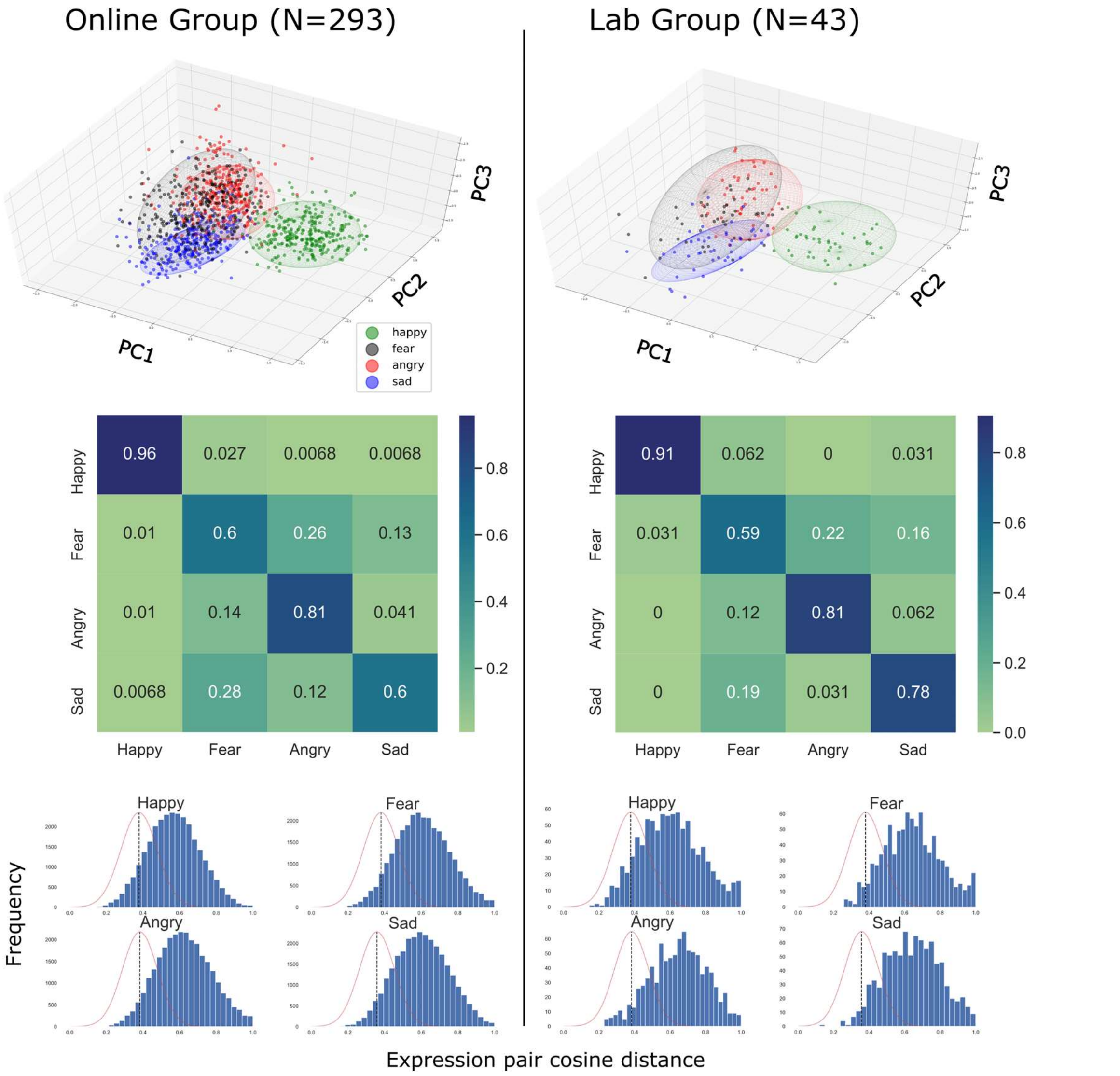

Fig S5: Final preferred expression positioning, clustering, and distances in expression space, for the Online and Lab groups separately. a) Dispersion of participants' preferred expressions across the first 3 Principal Components (PCs) of facial expression. Ellipsoids depict dispersion of expressions per emotion cluster, identified through Gaussian Mixture Model - GMM (radii scaled to encompass 2 standard deviations per PC. b) GMM confusion matrix depicting probability of expressions matching the corresponding cluster label, and characterizing cluster overlap based on classification rate. c) GA stochastic noise threshold (dotted orange lines) related to the distribution of differences (cosine distance in blendshape space) of all possible participant expression pairings per emotion category (blue histograms). Area of the blue curve above these noise thresholds identifies the % of participant expression pairings whose differences exceed variability explained by GA stochastic noise.

Comparison of emotion identification performance between GA and KDEF stimuli

Participants (N=60) who had not previously evolved GA expressions labelled (happy, fear, angry or sad) expressions belonging to either the GA stimulus set, or the Karolinska Directed Emotional Faces (KDEF) database (3) (Fig. S6). Rates of correct identification were submitted to a 2x4 repeated measures ANOVA, with factors Stimulus (GA / KDEF) and Emotion (happy / fear / angry / sad). Mauchly's test indicated that the assumption of sphericity was violated for Emotion ($\chi 2$=13.13, p=.02) and for the Stimulus x Emotion interaction ($\chi 2$=36.13, p<.001), therefore degrees of freedom were corrected using Huynh-Feldt estimates of sphericity (ε=.92 and ε=.8, respectively). We found a main effect of Stimulus (F(1,59)=158.29, p<.001, $\eta 2$=.1) of Emotion (F(2.78,164.23)=107.75, p<.001, $\eta 2$=.41), and a significant Stimulus x Emotion interaction (F(2.39,140.93)=37.21, p<.001, $\eta 2$=.08). Bonferroni corrected post-hoc comparisons (critical p=.01) showed that GA preferred expressions had lower rates of identification than KDEF stimuli for happy (t(59)=-3.19, p=.002), fear (t(59)=-6.2, p<.001) and sad (t(59)=-10.96, p<.001), but equivalent identification rates for angry expressions (t(59)=-1.27, p=.21; Fig 3d; full stats in table below). Taken together, while both types of stimuli show similar patterns of expression recognition per category, expressions portrayed by GA stimuli led to lower rates of identification at a group level (with the exception of angry expressions). We also collected a second group of 60 participants who performed the same task, but with GA and KDEF stimuli blocked separately. Results mimicked those of Group 1, with a main effect of Stimulus (F(1,59)=114.29, p<.001, $\eta 2$=.1) of Emotion (F(2.29,151.4)=68.8, p<.001, $\eta 2$=.38), and a significant Stimulus x Emotion interaction (F(2.56, 151.4)=9.93, p<.001, $\eta 2$=.02). Post-Hoc t-tests showed a significant difference between GA and KDEF for happy (t(59)=-4.13, p<.001), fear(t(59)=-6.9, p<.001) and sad (t(59)=-7.8, p<.001) expressions. However group 2 also showed a significant (but smaller) difference between GA and KDEF angry stimuli (t(59)=-2.7, p=.03)

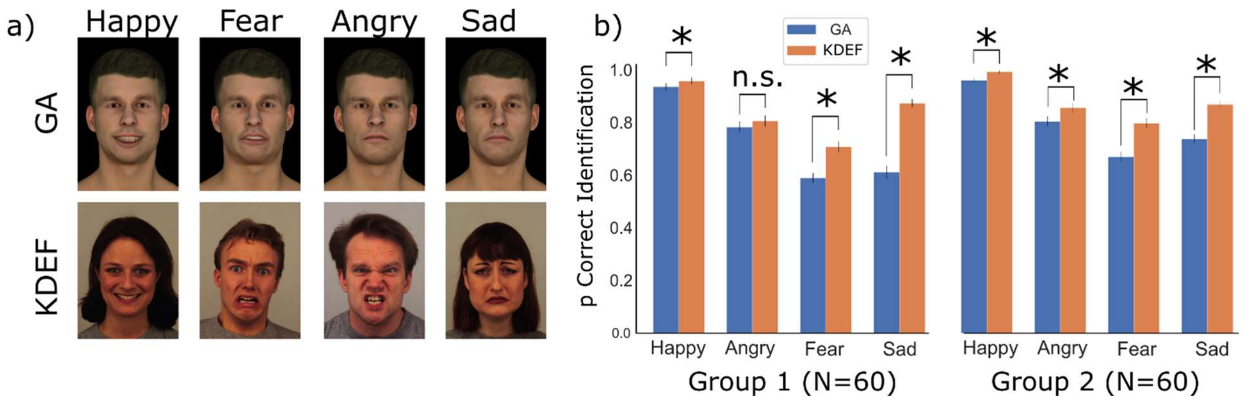

Fig. S6. Agreement across participants on emotion of GA evolved expressions. a) Participants labelled GA expressions evolved by a different group of participants (top row), or labelled posed expressions drawn from the Karolinska Directed Emotional Faces (KDEF) database (bottom row). b) Probability of correct expression identification as a function of emotion category and stimulus type tested in interleaved (left) or blocked (right) presentations. Error bars represent standard error of the mean (SEM).

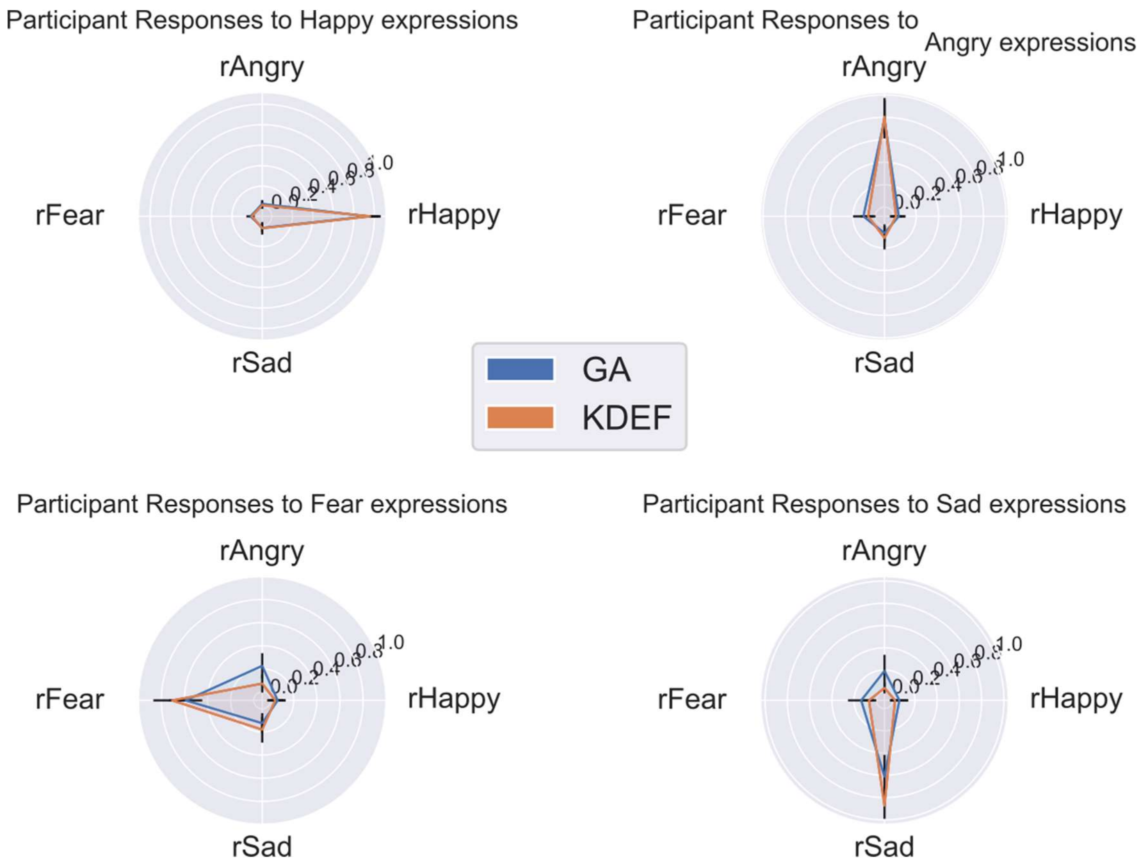

Fig. S7. External validation of GA evolved expressions. Polar plots depicting rates of participant response (rHappy, rFear, rAngry, rSad) based on stimulus expression category (happy, fear, angry, sad). Blue and Orange lines represent pooled performance using GA and KDEF stimuli, respectively. While there is significant overlap in response probabilities to both categories of stimuli, GA stimuli show greater rates of labelling errors, especially within fear and sad expressions. e.g. GA fear stimuli are more frequently confused as 'angry', with respect to KDEF fear stimuli. Happy expressions showed a complete overlap between GA and KDEF stimuli.

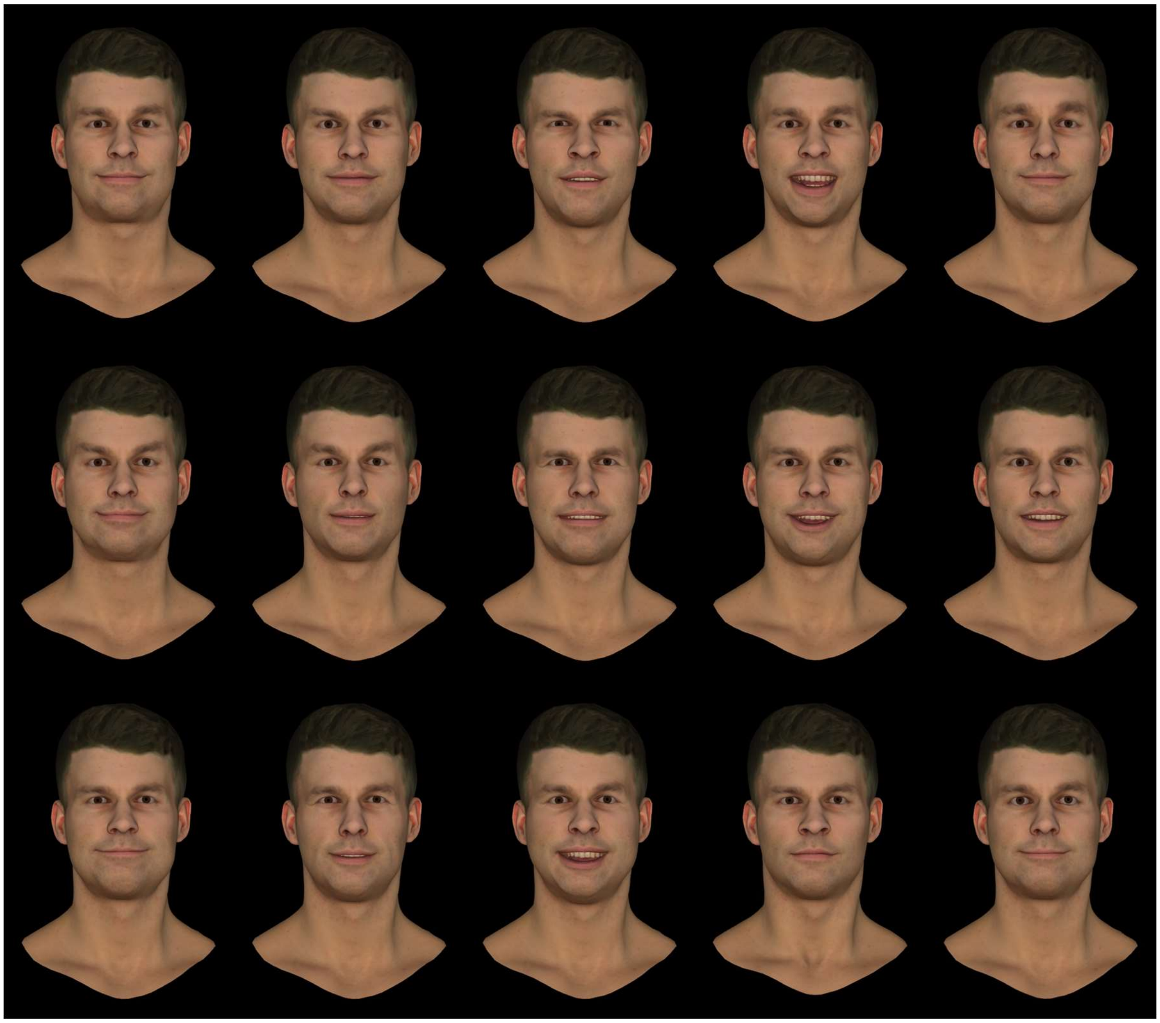

Fig. S8. Sample GA preferred expressions: 15 randomly sampled Happy expressions per emotion category

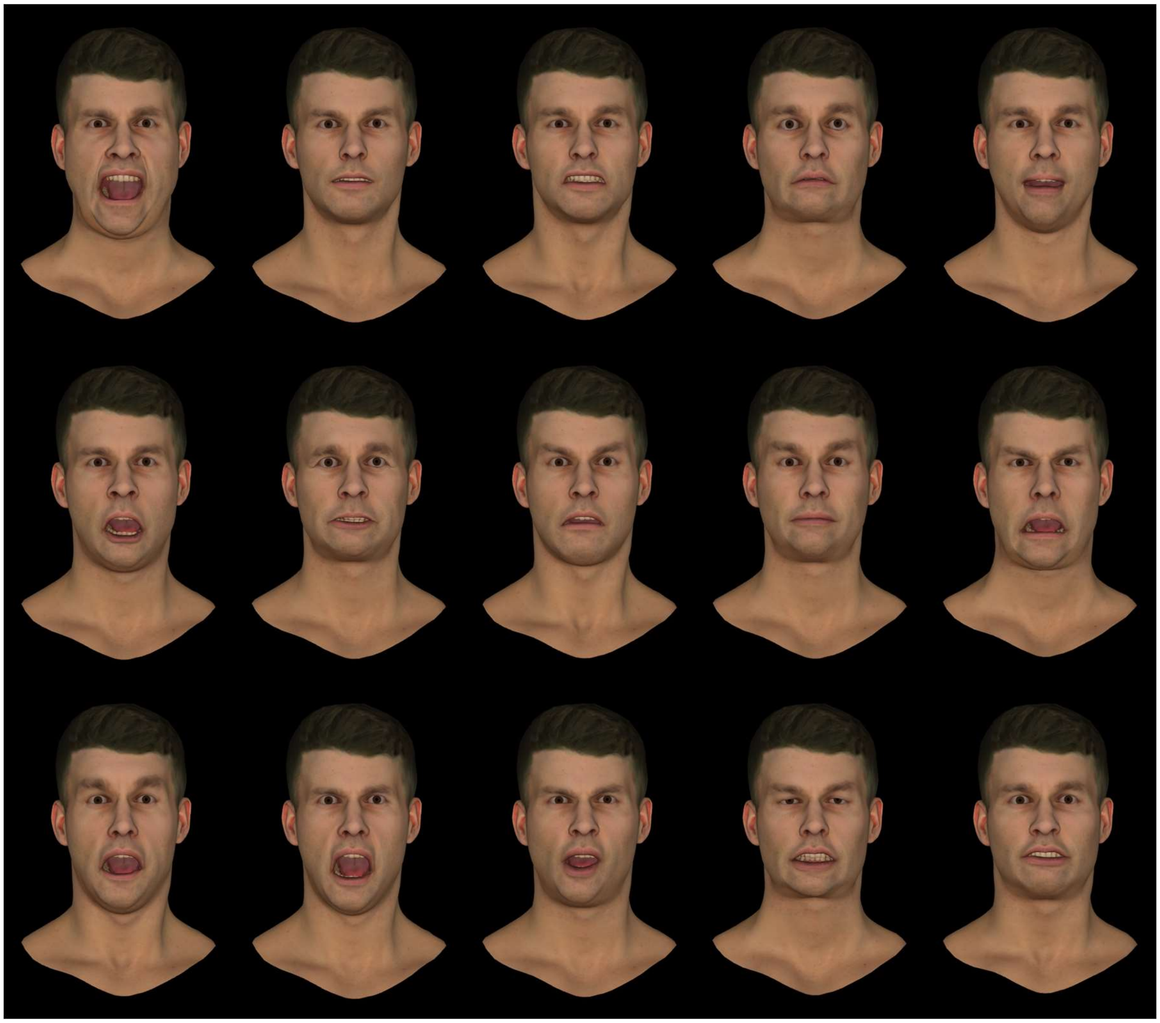

Fig. S9. Sample GA preferred expressions: 15 randomly sampled Fear expressions per emotion category (not the same participants within each emotion)

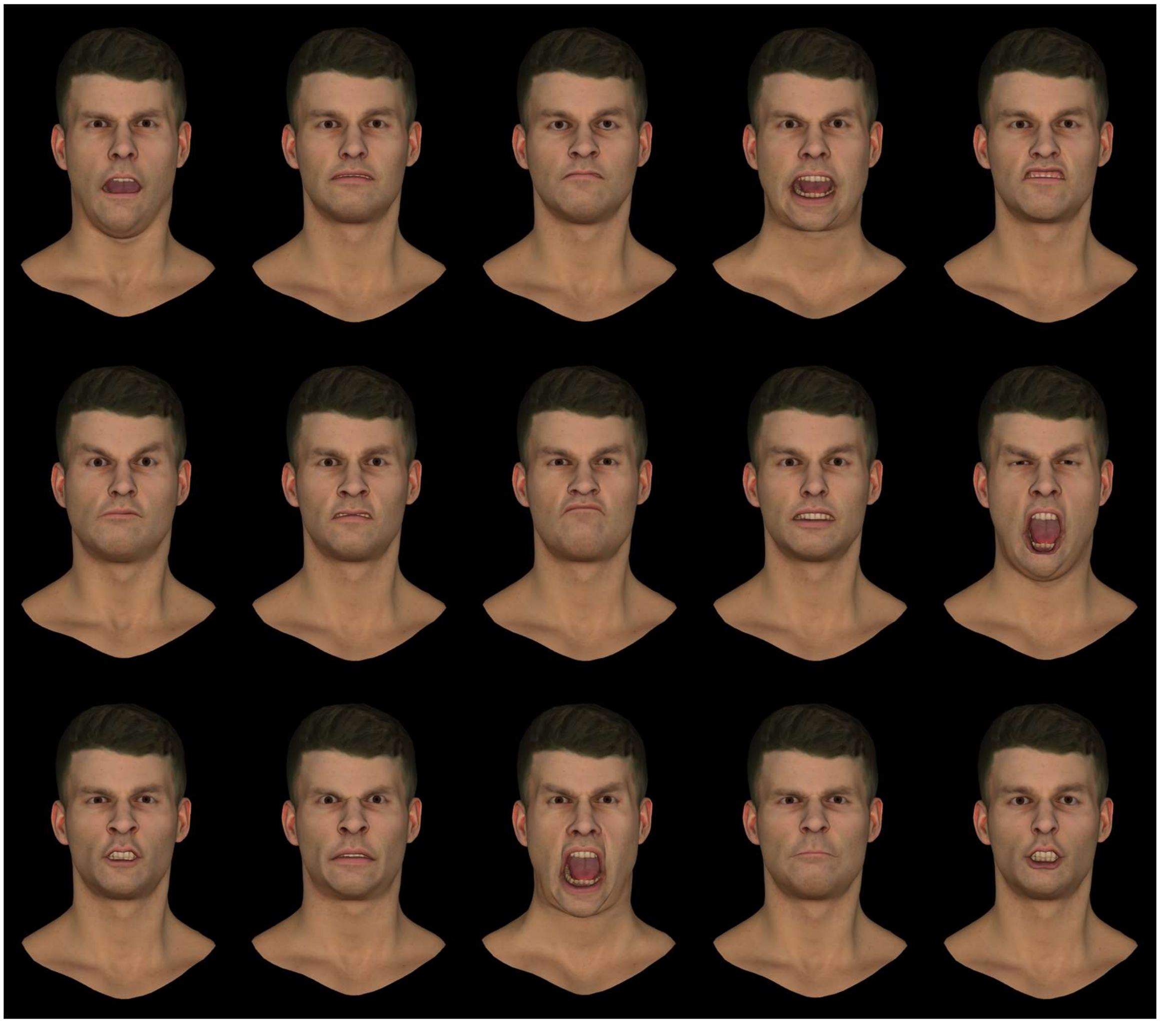

Fig. S10. Sample GA preferred expressions: 15 randomly sampled Angry expressions per emotion category (not the same participants within each emotion)

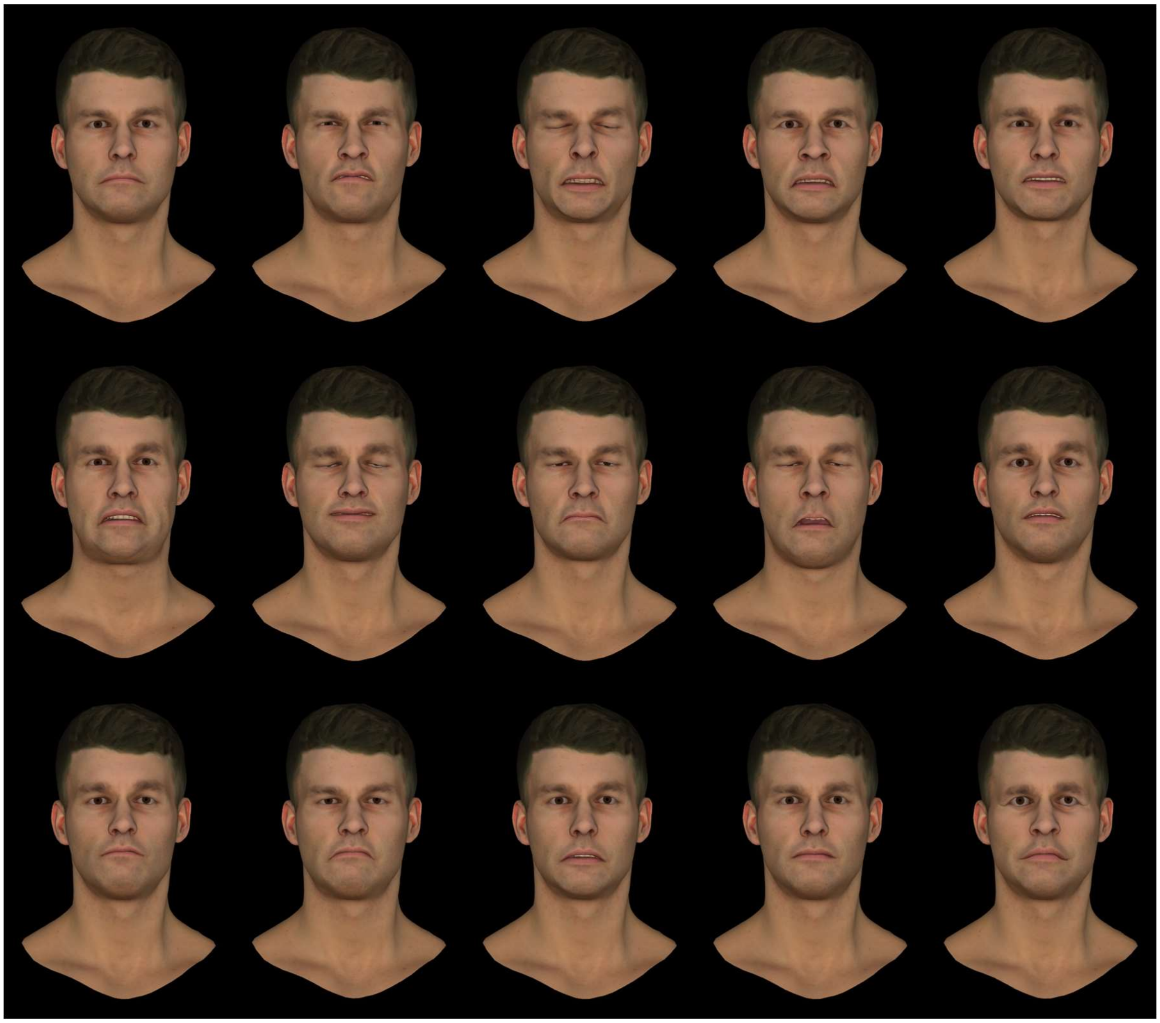

Fig. S11. Sample GA preferred expressions: 15 randomly sampled Sad expressions per emotion category (not the same participants within each emotion)

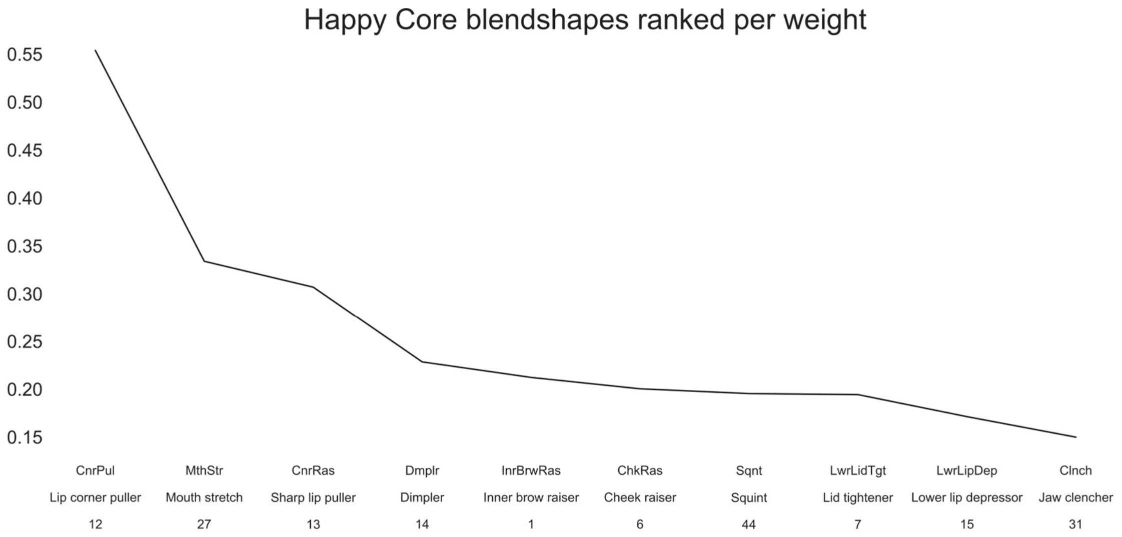

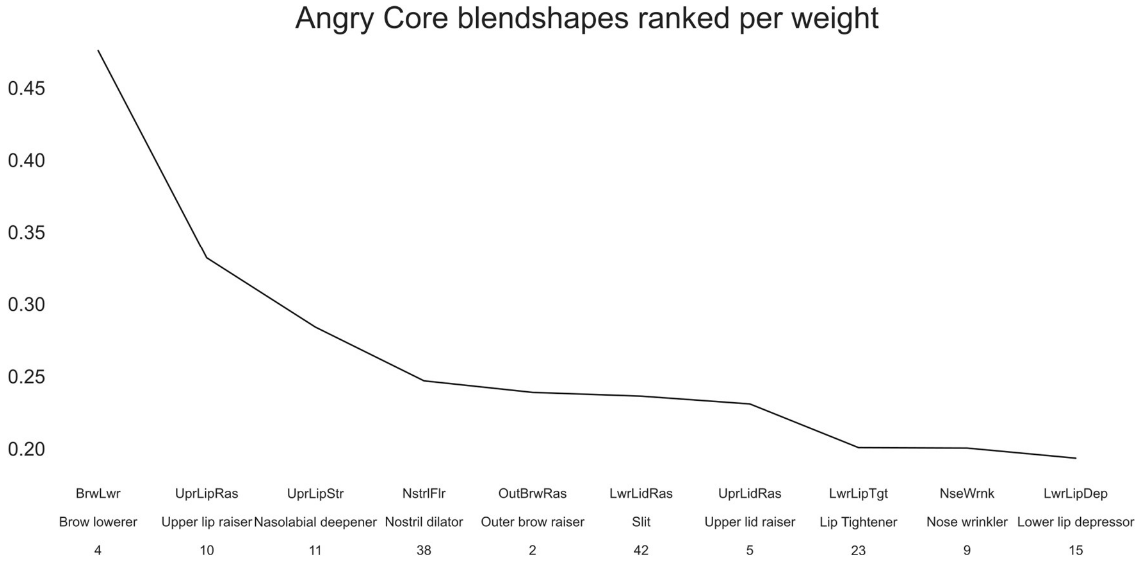

Fig. S12. Expression features that mostly contribute to Happy / Angry expression categories (i.e. are most activated in expressions belonging to these categories), ranked by blendshape weight. Blendshape weight can be thought of as the contraction of a muscle group, ranging from 0 - fully relaxed, to 1 - fully contracted. Ranking blendshapes based on activation permits us to determine which set of action units mostly contribute to a specific expression. However, the rank of these activations shouldn't be strictly interpreted as "order of importance". Two action units might be systematically present in a given expression, and one might be more pronounced than the other, but both could still contribute significantly to the expression . Each plot depicts the blendshape name, FACS code and Action Unit (AU) of the first 10 blendshapes (x-axis, stacked) ranked based on average blendshape weight value (y-axis).

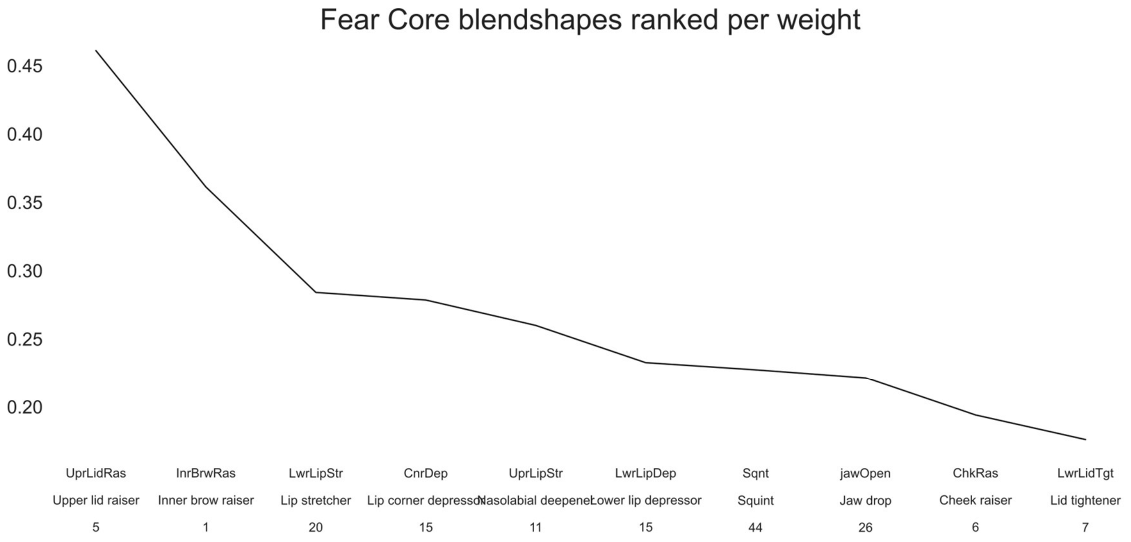

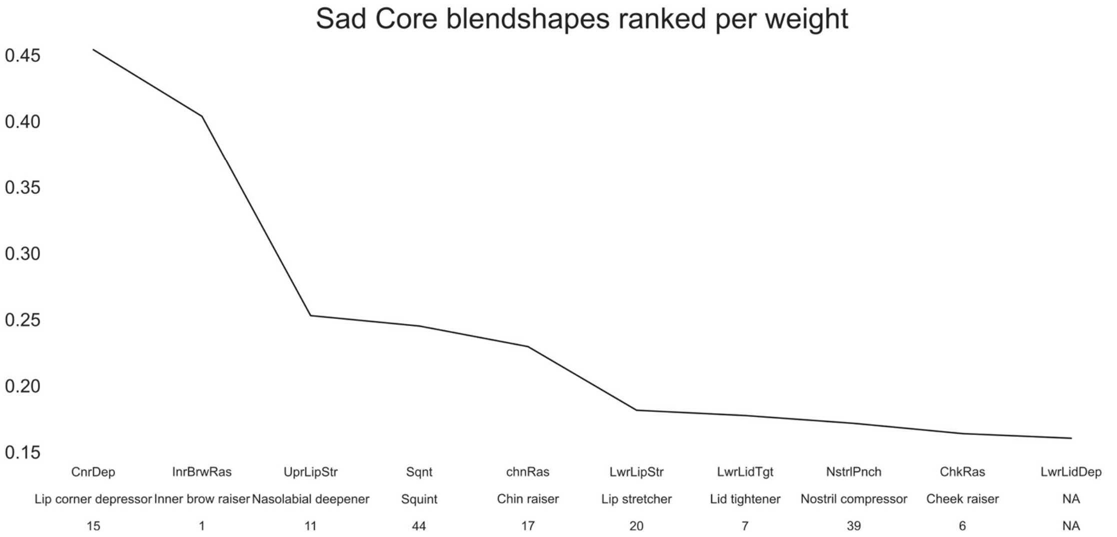

Fig. S13. Expression features that mostly contribute to Fear / Sad expression categories (i.e. are most activated in expressions belonging to these categories), ranked by blendshape weight. Each plot depicts the blendshape name, FACS code and Action Unit (AU) of the first 10 blendshapes (x-axis, stacked) ranked based on average blendshape weight value (y-axis).

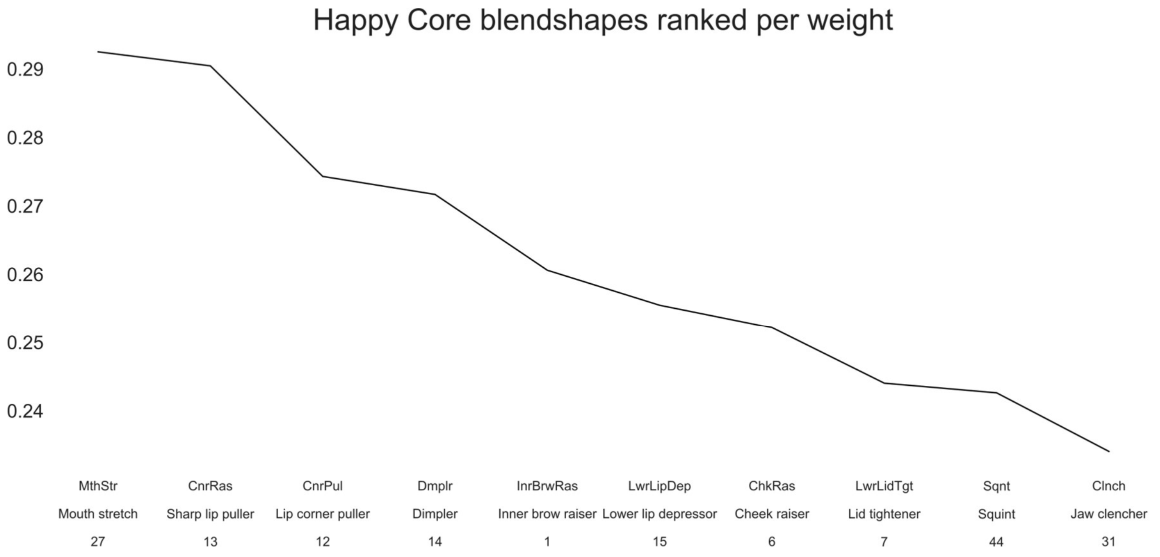

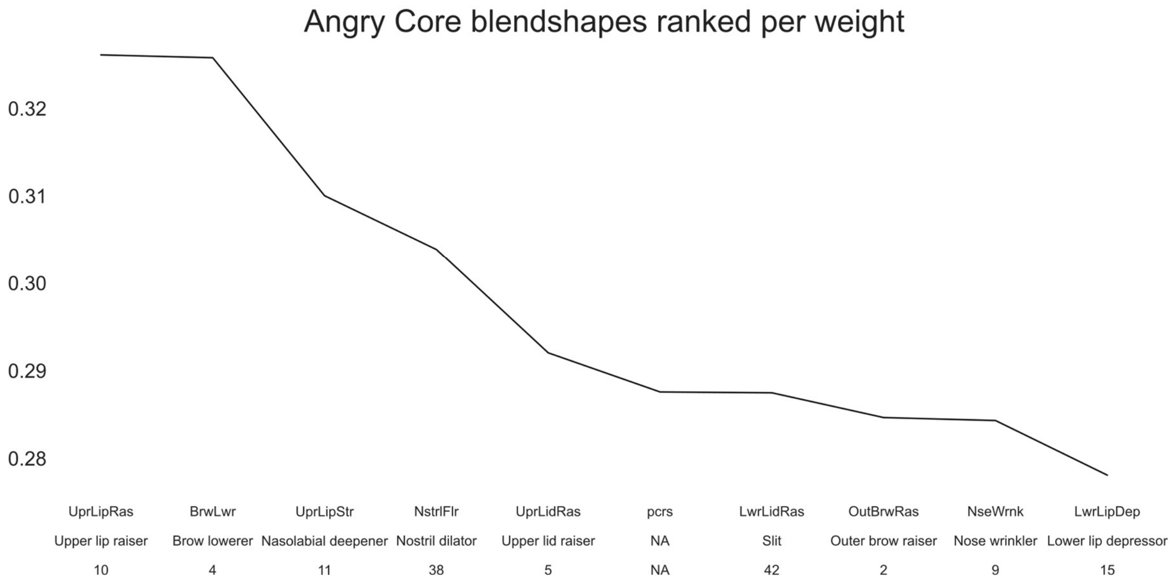

Fig. S14. Expression features that vary the most amongst across participants per Happy / Angry categories, ranked by blendshape weight variability (10 samples). Each plot depicts the blendshape name, FACS code and Action Unit (AU) of the first 10 blendshapes (x-axis, stacked) ranked based on average blendshape weight standard deviation (y-axis).

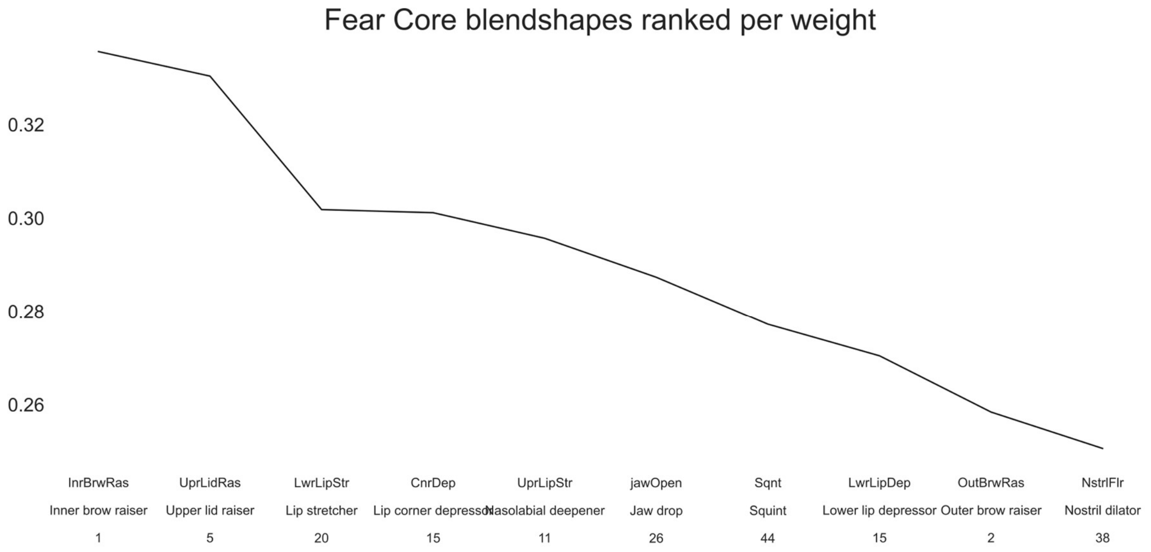

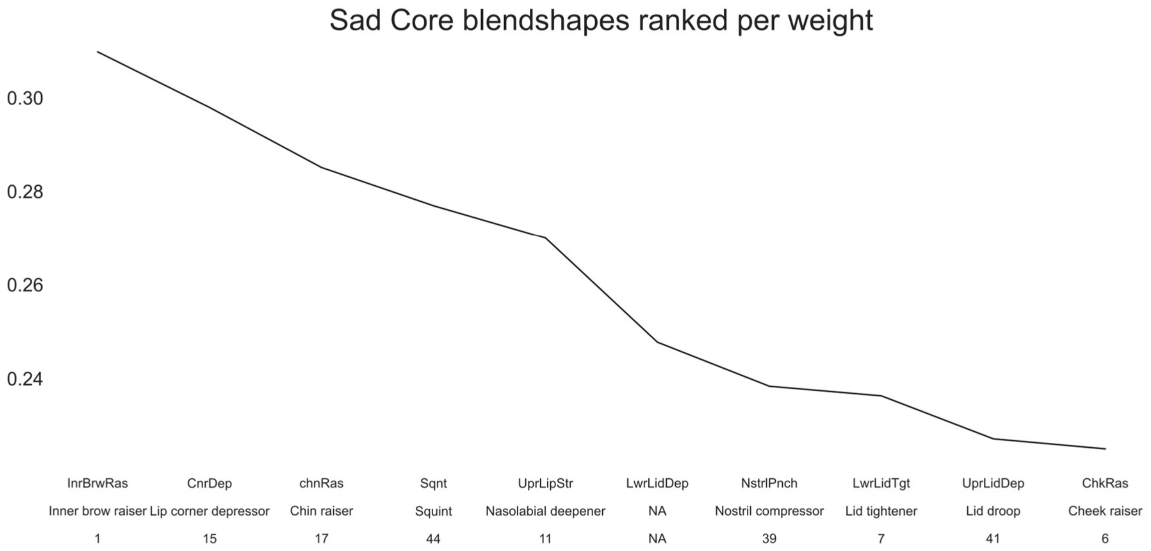

Fig. S15. Expression features that vary the most amongst across participants per Fear / Sad categories, ranked by blendshape weight variability (10 samples). Each plot depicts the blendshape name, FACS code and Action Unit (AU) of the first 10 blendshapes (x-axis, stacked) ranked based on average blendshape weight standard deviation (y-axis).

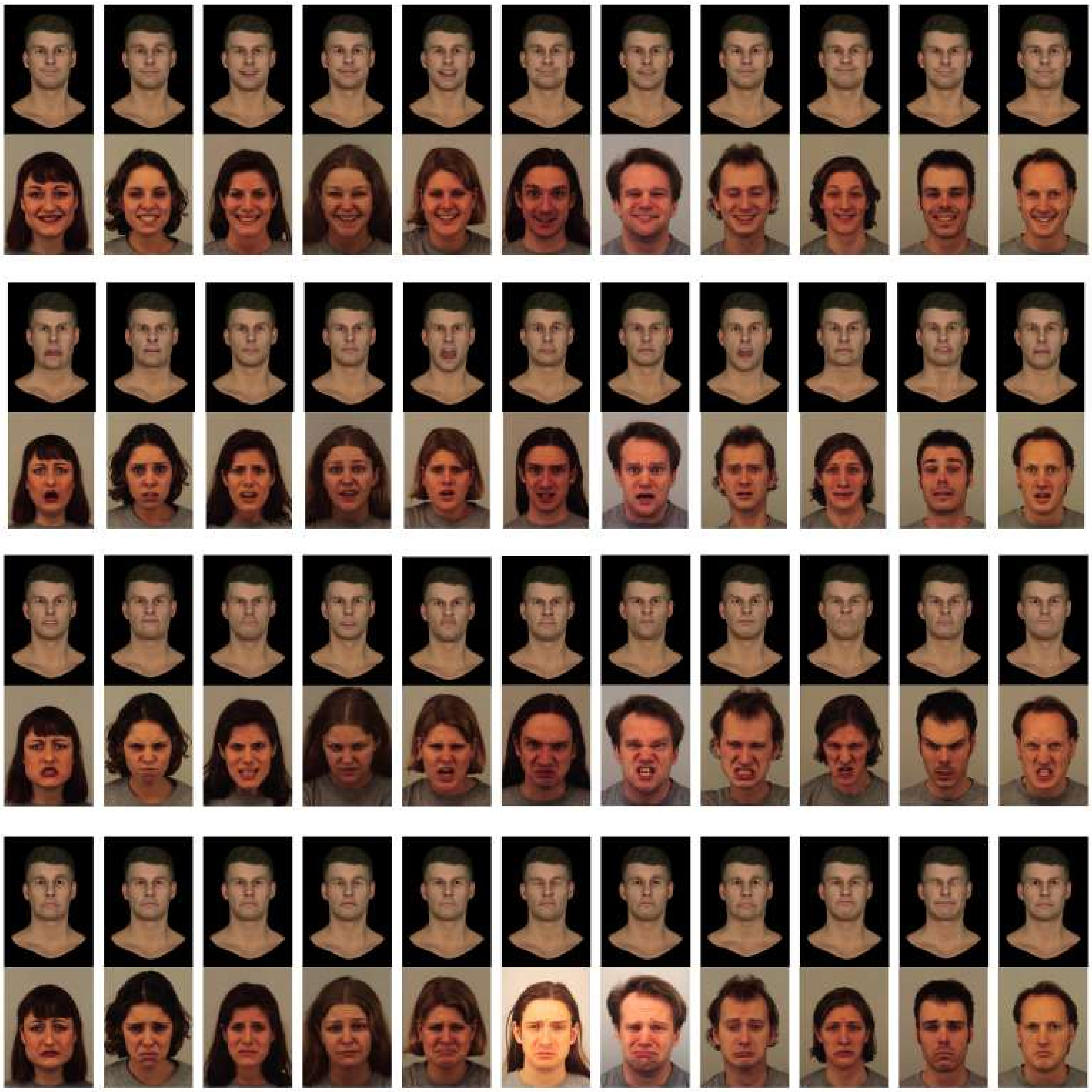

Fig. S16. GA and KDEF sample expressions per emotion category used to assess emotion recognition.

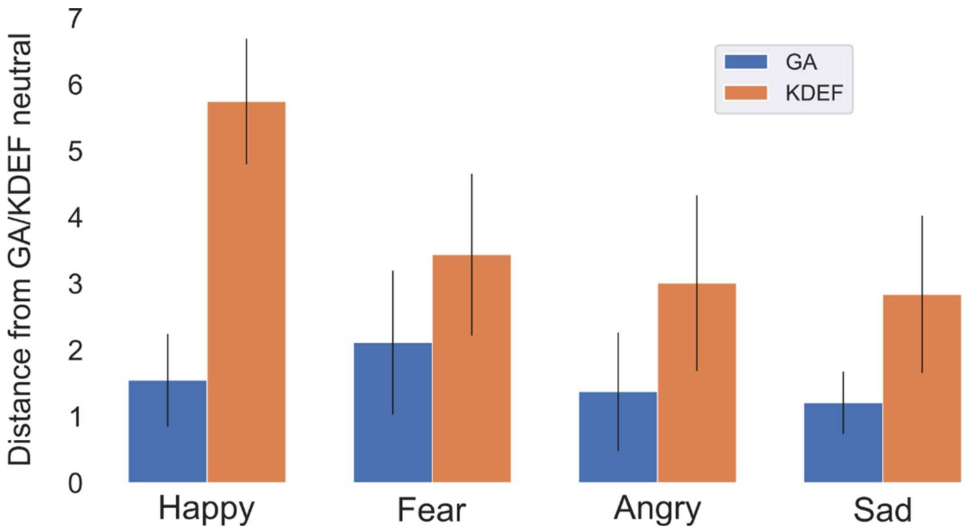

Fig. S17. GA and KDEF spread in expression space. We compared the spread of Happy/Fear/Angry/Sad GA and KDEF expressions within the same quantitative space. 293 GA evolved expressions and 70 KDEF stimuli portraying Happy/Fear/Angry/Sad and Neutral expressions were automatically FACS coded using the OpenFace software (https://github.com/TadasBaltrusaitis/OpenFace), yielding scores along 17 facial expression Action Units. We submitted these scores to Principal Component Analysis, representing GA and KDEF expressions in a 10 dimensional space and accounting for 75% of variance in the data. We then calculated the Euclidean distance in PCA space between each expression and its Neutral face, as a way of quantifying spread in PCA space factoring out identity differences. This involved calculating the distance between each GA expression and a constant Neutral GA stimulus, and the distance between each KDEF expression and the Neutral stimulus portrayed by the same actor. Barplots represent average distance of GA / KDEF stimuli relative to a Neutral expression. Greater distances of KDEF stimuli suggest greater intensity of KDEF expressions relative to GA expressions.

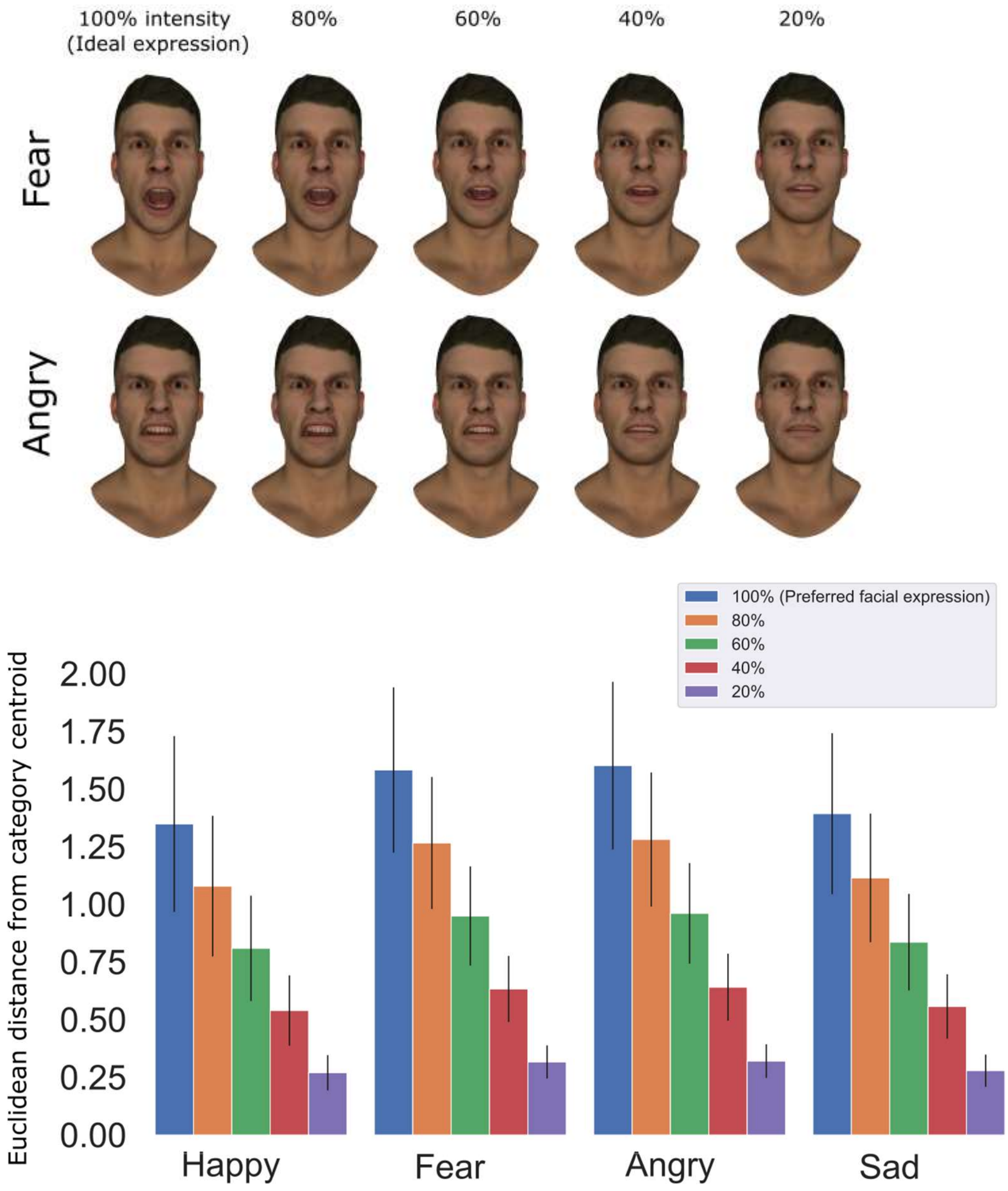

Fig. S18. Spread of expressions in expression space as a function of stimulus intensity. We parametrically manipulated the intensity of GA preferred expressions to characterize how expression intensity affects spread of expressions in PCA space. We scaled blendshape weights of 293 Happy/Fear/Angry and Sad Ideal expressions in 5 linearly spaced intervals: 100% (the original preferred expression), 80/60/40 and 20% scaled intensity intervals. We then calculated distance from Neutral, similarly to Fig S17. We observed that spread in PCA space linearly decreased as a function of intensity scaling percentage, showing that intensity affects spread of samples in expression space.

blendshapes are shown at the bottom of each emotion section. Although inspired by the FACS system, blendshapes don't fully map onto AUs. Some AUs aren't associated with a dedicated controller, but are expressed through a combination of blendshapes. This includes Lips Part (25) and Eyes Closed (43), which can be observed above for Happy and Sad expressions, respectively. While we lack dedicated controllers for these AUs, we can see in the 3D renders that participants indeed evolved Happy expressions with lips apart (Fig S8), and sad expressions with eyes closed (Fig S11). Despite the overlap in AUs across studies, comparisons also reveal variability in expressions based on stimuli, methods and tested populations. For instance we found Mouth Stretcher (27) and Squint (44) for Happy, Lip Corner Depressor (15), Nasolabial Deepener (11) and Lower Lip Depressor (16) for Fear, Upper Lip Raiser (10), Nasolabial Deepener (11), and Outer Brow

Raiser (2) for Angry, and Nasolabial Deepener (11), Squint (44) and Lip Stretcher (20) for Sad expressions, which were not documented in the reviewed literature. Several participants evolved Happy expressions with open mouths, which might explain AU 27 (although Lips Part AU25, and Jaw Drop AU26, associated with more subtle mouth opening movements have been observed in the reviewed studies). Also, Lip Corner Depressor (15), which is typically reported for Sadness, was also highly activated in participants' Fear expressions (and not observed in other reviewed studies), highlighting the overlap between these two categories. This, together with the variability that can also be observed amongst these reviewed studies, highlight the variability of expression beyond core emotion descriptors identified in the Ekman & Friesen classification.

Expression discrimination task

Given the variability in preferred facial expressions, we expect that participants will also vary in their perceptual sensitivity to differences in facial expressions. We examined this in a different subsample of participants (N=62, M=40, F=22, age= 28.4+/-9) who had previously evolved preferred facial expressions using the GA toolkit. We created continua of expression stimuli between 2 negative preferred facial expressions (e.g. "Angry" to "Fear" continuum) and participants were tested in random pairs, with each pair member contributing one continuum of stimuli based on their evolved expressions. Participants were recruited through the Prolific online platform. After reading the information sheet and providing informed consent, participants were redirected to an emotion recognition task hosted on Pavlovia (https://pavlovia.org/). Stimulus presentation conditions were identical to the GA validation and GA expression Categorization experiments.

Continua were created by rendering new facial images from the GA preferred expressions (e.g. "Angry" and "Fear", in 7 linearly spaced steps). For example, the 1st and 7th images corresponded to a participant's unaltered Fear and Angry preferred facial expressions respectively, whereas the central (4th) step corresponded to a 50% blend of Fear and Angry expressions. Each pair was tested along one of three possible emotion axes: Fear-Angry, Fear-Sad or Angry-Sad. On every trial each member of the pair was randomly presented with an expression drawn from either their own continuum ("Participant") or the other's continuum ("Control"), and were asked to determine which category it belonged to, using a two alternative forced choice (e.g. Fear or Angry?). Note that while the continua tested were the same within each pair, the "Participant" and "Control" labels were swapped between pair members (i.e. one member's "Participant" continuum corresponded to the other's "Control" continuum, and vice versa).

We looked at participants' responses as a function of stimulus level, separately along the Participant and Control continua. We hypothesized that participants would be more sensitive to changes in expressions when tested with stimuli drawn from their own continuum (Participant continuum), since changes in the stimuli would occur on expressions that are unique to the participant (i.e. that were their preferred expressions). Greater sensitivity to differences in expression would result in a greater rate of change of responses across continuum steps (steeper slope in the Participant continuum responses). Each pair was tested on one of the three emotion continua (randomly selected), and we only analyzed pairs where both members were able to successfully perform the task on both continua (i.e. where their responses were captured by a sigmoid function with y at the leftmost step <= .25, and y at rightmost step >= .75). This resulted in 16 participants (8 pairs) on the Fear-Angry continua, 28 participants (14 pairs) on the Fear-Sad continua, and 18 participants (9 pairs) on the Angry-Sad continua. Each participant performed 20 trials per step (7) x 2 continua (280 trials in total). Pooled data fits support our hypothesis of a steeper slope (greater sensitivity to changes in expressions) within the Participant continua. We tested differences in slope steepness with a Mixed Effects Probit Regression, with Continuum (Participant / Control) and Emotions (Fear-Angry, Fear-Sad, Angry-Sad) as factors, and emotion Steps (1-7) as a covariate, participant as a Cluster variable, and Responses as the dependent variable. Participants were added as random intercept. This analysis revealed a significant Continuum*Steps interaction (Wald test $\chi^2(1)= 59.14$, $p<.001$), indicating a steeper slope in the Participant relative to the Control continua by a factor of 1.14 ($z = 7.7$, $p<.001$). Analysis were carried out on the Jamovi statistical software (https://www.jamovi.org/), using the Generalized Mixed Models module of the GAMLj suite (https://gamlj.github.io/gzlmmixed.html).

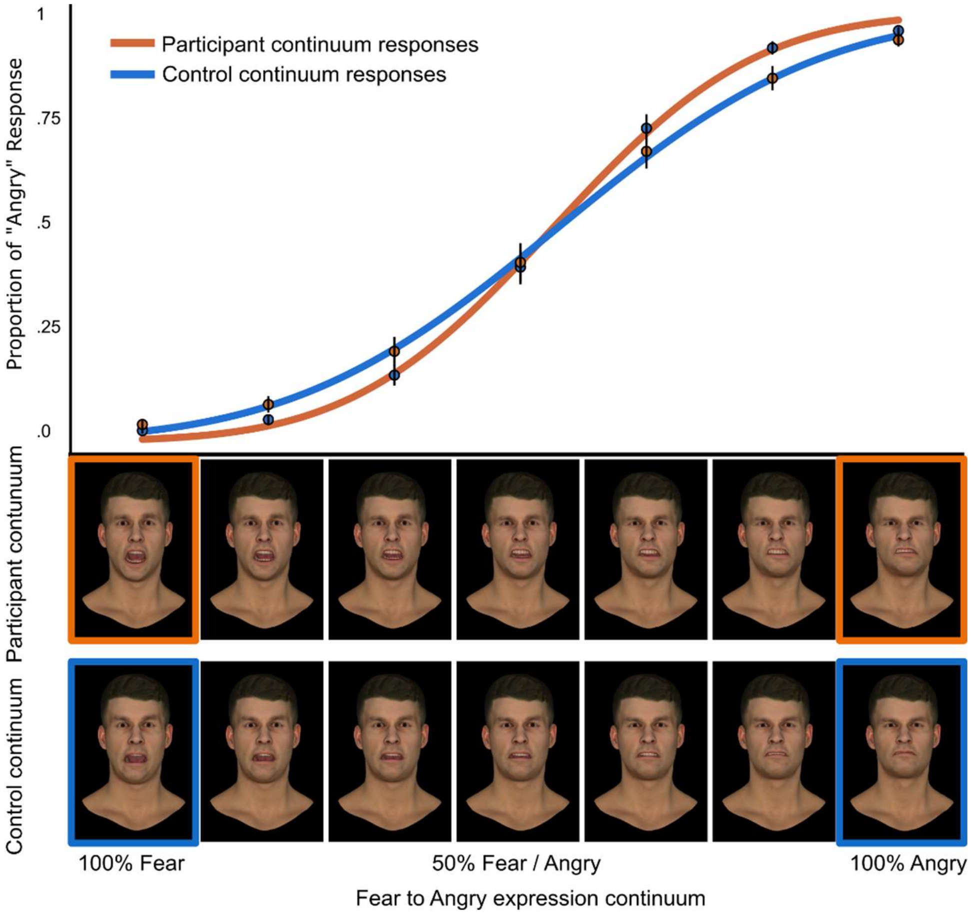

Fig. S19: Responses pooled across all participants and all emotion combinations, fit with a cumulative Gaussian function. Participants were randomly paired, with each pair member contributing one continuum of stimuli connecting his/her GA evolved expressions of two negative emotions (e.g. stimuli shown from Fear-Angry continua; note however that data depicts responses pooled across Fear-Angry, Fear-Sad, and Angry-Sad emotion combinations). Each pair member then performed an emotion classification task on stimuli drawn from either the continuum connecting his/her GA evolved expressions ("Participant") or the continuum connecting the other member's GA evolved expressions ("Control"). Separate groups of participants were tested on different emotion combinations. Plots show change in participant responses (proportion of one emotion type response, (e.g. "Angry") as a function of stimulus level along "Participant" and "Control" continua. Plots show a steeper change in classification responses when participants are presented stimuli drawn from a continuum connecting the two preferred facial expressions they had previously evolved.

			Posed		Spontaneous		Data Driven	
	GA expressions (top 15 AUs)		Kohler et al., 2004	Cordaro et al., 2018	BP4D - Zhang et al., 2014	CK+ - Lucey et al., 2010	Jack et al., 2016	Yu et al., 2012
	Facs Name	AU	Facs Name	Facs Name	Facs Name	Facs Name	Facs Name	Facs Name
Happy	Lip corner puller	12	x	x	x	x	x	x
	Mouth stretch	27						
	Sharp lip puller	13					x	x
	Dimpler	14					x	x
	Inner brow raiser	1	x				x	
	Cheek raiser	6	x	x	x		x	x
	Squint	44						
	Lid tightener	7	x	x				
	Lip corner depressor	15						
	Jaw clencher	31						
	Lip suck	28						
	Outer brow raiser	2						
	Nasolabial deepener	11						
	Nostril dilator	38					x	
	Slit	42						
			Jaw drop (26)	Lower lip depressor (16)			Lips part (25)	Lips part (25)
			Upper lip raiser (10)	Lips part (25)				
			Lips part (25)					
Fear	Upper lid raiser	5	x	x	x	x	x	x
	Inner brow raiser	1	x	x	x	x	x	
	Lip stretcher	20	x					x
	Lip corner depressor	15						
	Nasolabial deepener	11						
	Lower Lip Depressor	16						
	Squint	44						
	Jaw drop	26	x				x	
	Cheek raiser	6						
	Lid tightener	7		x				
	Outer brow raiser	2	x	x	x	x	x	
	Nostril dilator	38						
	Nostril compressor	39						
	Brow lowerer	4	x		x	x		
	Jaw clencher	31						
				Lips part (25)			Mouth stretch (27)	
							Lips part (25)	
Angry	Brow lowerer	4	x	x				
	Upper lip raiser	10	x				x	x
	Nasolabial deepener	11					x	
	Nostril dilator	38						
	Outer brow raiser	2						
	Slit	42						
	Upper lid raiser	5	x					
	Lip Tightener	23						
	Nose wrinkler	9	x				x	x
	Lower lip depressor	16	x				x	x
	Lip corner depressor	15						
	Lid tightener	7	x	x			x	x
	Lip Tightener	23			x	x		
	Lip suck	28						
	Lip Funneler	22					x	
			Lips part (25)		Lip pressor (24)	Lip pressor (24)		Lips part (25)
			Lip stretcher (20)					
			Jaw drop (26)					
Sad	Lip corner depressor	15	x		x	x	x	x
	Inner brow raiser	1	x					
	Nasolabial deepener	11			x	x		
	Squint	44						
	Chin raiser	17	x				x	x
	Lip stretcher	20	x				x	
	Lid tightener	7	x		x	x	x	
	Nostril compressor	39					x	x
	Cheek raiser	6			x	x	x	
	Jaw clencher	31						
	Lid droop	41						
	Jaw thrust	29						
	Slit	42						
	Nostril dilator	38						
	Brow lowerer	4	x	x	x	x	x	x
			Upper lip raiser (10)	Eyes closed (43)			Eyes closed (43)	Lip tightener (23)
			Lips part (25)	Head down (54)			Lip pressor (24)	Eyes closed (43)

Table S1: Table showing the top 15 activated blendshapes / Action Units (AU) in averaged happy, fear, angry and sad GA preferred expressions, compared to expression descriptors documented in (4–9). AUs reported in these studies that are not listed amongst our top 15 active

SI References

1. N. Roubtsova, *et al.*, EmoGen: Quantifiable Emotion Generation and Analysis for Experimental Psychology. *arXiv:2107.00480 [cs]* (2021) (July 20, 2021).

2. C. Carlisi, *et al.*, Using evolutionary algorithms to uncover individual differences in how humans represent facial emotion (2020) https:/doi.org/10.31234/osf.io/9fta2 (June 16, 2021).

3. D. Lundqvist, A. Flykt, A. Öhman, The Karolinska directed emotional faces (KDEF). *CD ROM from Department of Clinical Neuroscience, Psychology section, Karolinska Institutet* **91**, 2–2 (1998).

4. C. G. Kohler, *et al.*, Differences in facial expressions of four universal emotions. *Psychiatry Research* **128**, 235–244 (2004).

5. D. T. Cordaro, *et al.*, Universals and cultural variations in 22 emotional expressions across five cultures. *Emotion* **18**, 75–93 (2018).

6. X. Zhang, *et al.*, BP4D-Spontaneous: a high-resolution spontaneous 3D dynamic facial expression database. *Image and Vision Computing* **32**, 692–706 (2014).

7. P. Lucey, *et al.*, The Extended Cohn-Kanade Dataset (CK+): A complete dataset for action unit and emotion-specified expression in *2010 IEEE Computer Society Conference on Computer Vision and Pattern Recognition - Workshops*, (2010), pp. 94–101.

8. R. E. Jack, W. Sun, I. Delis, O. G. B. Garrod, P. G. Schyns, Four not six: Revealing culturally common facial expressions of emotion. *Journal of Experimental Psychology: General* **145**, 708–730 (2016).

9. H. Yu, O. G. B. Garrod, P. G. Schyns, Perception-driven facial expression synthesis. *Computers & Graphics* **36**, 152–162 (2012).
